# Unveiling the Causes of Acute and Non-Acute Myocardial Ischemic Syndromes: The Role of Optical Coherence Tomography

**DOI:** 10.3390/medicina61071218

**Published:** 2025-07-04

**Authors:** Angela Buonpane, Alberto Ranieri De Caterina, Giancarlo Trimarchi, Francesca Maria Di Muro, Domenico Galante, Samuela Zella, Fausto Pizzino, Marco Ciardetti, Umberto Paradossi, Giovanni Concistrè, Sergio Berti, Antonio Maria Leone, Filippo Crea, Carlo Trani, Francesco Burzotta

**Affiliations:** 1 Ospedale Isola Tiberina-Gemelli Isola, 00186 Rome, Italy; buonpaneangela@gmail.com (A.B.); domenico.galante1991@gmail.com (D.G.); antoniomaria.leone@fbf-isola.it (A.M.L.); filippo.crea@fbf-isola.it (F.C.); 2 Fondazione Toscana G. Monasterio, Ospedale del Cuore G, Pasquinucci, 54100 Massa, Italy; adecaterina@ftgm.it (A.R.D.C.); fpizzino@ftgm.it (F.P.); mciard@ftgm.it (M.C.); uparadossi@ftgm.it (U.P.); ifcberti@ftgm.it (S.B.); 3 Interdisciplinary Center for Health Sciences, Scuola Superiore Sant’Anna, 56127 Pisa, Italy; 4 Department of Medicine, Surgery and Dentistry, University of Salerno, Baronissi, Salvador Allende Street 43, 84081 Salerno, Italy; fdimuro94@gmail.com; 5 IRCCS Ospedale Policlinico San Martino, 16132 Genoa, Italy; s.zella89@gmail.com; 6 Department of Adult Cardiac Surgery, Ospedale del Cuore G, Pasquinucci, 54100 Massa, Italy; concistr@ftgm.it; 7 Department of Cardiovascular Sciences, Fondazione Policlinico Universitario A. Gemelli IRCCS, Università Cattolica Sacro Cuore, Largo Agostino Gemelli, 1, 00168 Rome, Italy; carlo.trani@unicatt.it (C.T.); francesco.burzotta@unicatt.it (F.B.)

**Keywords:** Optical Coherence Tomography, Acute Myocardial Ischemic Syndromes, Non-Acute Myocardial Ischemic Syndromes, tailored therapy, precision medicine

## Abstract

Despite significant advances in understanding and management, cardiovascular diseases remain the leading cause of mortality worldwide. Historically, diagnostic and therapeutic strategies have typically targeted obstructive coronary arteries. However, growing evidence supports the pivotal role of non-obstructive mechanisms in myocardial ischemia, prompting a new classification that distinguishes Acute Myocardial Ischemic Syndromes from Non-Acute Myocardial Ischemic Syndromes. In this evolving context, Optical Coherence Tomography (OCT) plays an important diagnostic role in the assessment of both obstructive and non-obstructive ischemic mechanisms. In Acute Myocardial Ischemic Syndromes, OCT enables the identification of major plaque destabilization mechanisms and contributes to the diagnosis of Myocardial Infarction with Non-Obstructive Coronary Arteries, helping to differentiate between atherosclerotic and non-atherosclerotic causes. In Non-Acute Myocardial Ischemic Syndromes, OCT assists in evaluating stenosis severity, plaque morphology, vulnerability, and healing, and may contribute to the diagnosis of Ischemia with Non-Obstructive Coronary Arteries, identifying myocardial bridge and epicardial spasm alongside conventional functional assessment of intermediate stenoses. This narrative review outlines the expanding clinical applications of OCT across the full spectrum of ischemic syndromes, emphasizing its role in bridging obstructive and non-obstructive pathophysiology and supporting a more comprehensive diagnostic approach to ischemic heart disease.

## 1. Introduction

*The one who leaves the cave and removes the blindfold from his eyes begins to see.*

*And in seeing, he comes to understand.*

*And in understanding, he may marvel at the light and beauty he no longer dared to believe in.*

*-Allegory of the Cave, Plato (Republic, Book VII)*

Despite significant scientific advances in understanding pathophysiology and managing known risk factors, cardiovascular diseases remain the leading cause of mortality and morbidity worldwide [1]. Traditionally, atherosclerotic obstructions in the epicardial coronary arteries have been considered as the main cause of myocardial ischemia, guiding both diagnostic and therapeutic approaches. However, with a deeper understanding of myocardial ischemic mechanisms, it has become evident that non-obstructive mechanisms also contribute significantly to ischemic syndromes. This paradigm shift has led to the development of a new classification system, distinguishing between Acute Myocardial Ischemic Syndromes (AMIS) and Non-Acute Myocardial Ischemic Syndromes (NAMIS) [2]. This revised framework integrates both obstructive and non-obstructive mechanisms, ensuring a more comprehensive and clinically relevant model for evaluating and managing ischemic heart disease.

In this evolving landscape, Optical Coherence Tomography (OCT) has emerged as a powerful intravascular imaging tool, extending beyond its traditional role in assessing epicardial coronary artery disease (CAD) [3,4]. Initially, OCT was primarily used to clarify ambiguous angiographic findings, overcome the limitations of two-dimensional coronary angiography, identify the mechanisms underlying acute coronary syndromes (ACS) [5], and guide percutaneous coronary interventions (PCI) [6]. However, its role has expanded significantly over time, proving to be essential in diagnosing myocardial ischemia even when significant coronary stenosis is not present. OCT provides high-resolution imaging, enabling the detection of crucial pathological features in both obstructive and non-obstructive ischemic syndromes. In AMIS, OCT can reveal plaque rupture (PR), plaque erosion (PE), and eruptive calcified nodules (CNs), helping to clarify the mechanisms underlying ACS [5]. In Myocardial Infarction with Non-Obstructive Coronary Arteries (MINOCA), OCT can differentiate between atherosclerotic causes, such as PR, PE, and CNs in non-obstructive coronary arteries, and non-atherosclerotic causes, including spontaneous coronary artery dissection (SCAD), epicardial coronary spasm (particularly when assessed with acetylcholine testing), and coronary embolism [7]. In NAMIS, OCT plays a pivotal role in assessing ischemia caused by epicardial coronary stenoses, evaluating plaque morphology and stenosis severity [3,4], while also providing insights into plaque vulnerability [8] and plaque healing [9,10]. Furthermore, in Ischemia with Non-Obstructive Coronary Arteries (INOCA), OCT can detect epicardial coronary spasm [11] and myocardial bridges (MB) [12], conditions that may otherwise remain undiagnosed with conventional coronary angiography.

This narrative review aims to explore the expanding role of OCT in both AMIS and NAMIS, emphasizing its utility not only in the assessment of obstructive coronary artery disease (CAD) but also in the detection and characterization of non-obstructive ischemic mechanisms, highlighting the transformative role of OCT in modern cardiovascular medicine. To ensure a comprehensive and up-to-date synthesis, we performed a targeted literature search using PubMed, Scopus, and Google Scholar databases, focusing on publications from 2000 to March 2024. Search terms included “Optical Coherence Tomography”, “OCT”, “myocardial ischemia”, “MINOCA”, “INOCA”, “plaque rupture”, and “plaque erosion”. Peer-reviewed original articles, clinical trials, systematic reviews, and position papers published in English were included. Although not conducted as a systematic review, our aim was to provide a clinically meaningful narrative based on current evidence regarding the diagnostic applications of OCT in ischemic heart disease, with the ultimate goal of bridging the gap between obstructive and non-obstructive ischemic conditions.

## 2. Technical Principles and Clinical Role of OCT: Strengths and Limitations in Coronary Artery Disease Assessment

OCT is a cutting-edge intravascular imaging technique that shares key features with both intravascular ultrasound (IVUS) and conventional microscopy. OCT and IVUS utilize comparable mechanisms for image generation, as both rely on detecting the amplitude and time delay of signals reflected and refracted by biological tissues—OCT using light and IVUS using sound [3]. The core distinction between them lies in the type of energy source: OCT employs near-infrared light, whereas IVUS operates with high-frequency sound waves. This fundamental difference leads to distinct imaging characteristics. Light travels significantly faster than sound (approximately 300,000 km/s versus 1500 m/s), and its much shorter wavelength enables higher resolution. Consequently, OCT offers excellent spatial resolution (between 1 and 20 µm) with moderate tissue penetration (~2 mm), while IVUS, though capable of deeper penetration, provides lower resolution (typically 0.1 to 1 mm, depending on the frequency used) [3,13]. OCT is capable of producing detailed axial, cross-sectional, and even 3D images. Axial scans are generated by measuring how long it takes for light to reflect off tissue structures, while multiple A-scans are compiled into B-scans, which represent cross-sectional views. By stacking B-scans, a three-dimensional reconstruction (3D-OCT) can be rendered. These images are then digitally processed and color-coded for visualization. The resemblance between OCT and microscopy lies in the quality of the images produced—OCT scans closely resemble those of histological sections. However, unlike traditional microscopy, which requires excised tissue samples and offers ultra-high resolution (≤1 µm) with limited penetration depth, OCT captures high-resolution images in vivo and in real time, avoiding the need for biopsies [3].

At the heart of OCT technology is low-coherence interferometry. This method allows for precise measurement of tissue structures by comparing light reflected from the tissue with light from a reference arm. A basic OCT setup uses a Michelson interferometer, where a beam of light is split into two paths: a reference beam and a sample beam. After reflecting off their respective targets, the two beams are recombined. Their interference pattern, dependent on the optical path length difference, provides information about the depth and structure of the tissue. Constructive or destructive interference occurs depending on whether the electromagnetic waves are in or out of phase. Only when the path length difference is within the coherence length of the light source—meaning the waves are sufficiently synchronized—will interference be detected. This requires the light source to have low coherence, ensuring that only reflections from specific depths produce measurable interference [14].

Initially introduced in ophthalmology in the early 1990s with the first ex vivo retinal scan performed in 1991 [15], OCT gained traction through validation studies comparing its results with histological findings in both animal and human tissues. Early ophthalmic OCT systems used 800 nm light, but shifting to 1300 nm allowed for deeper tissue imaging, broadening its potential for other medical fields. The application of OCT in cardiovascular imaging was first explored by Brezinski and colleagues in 1995, who demonstrated its ability to capture the microstructural features of atherosclerotic plaques with impressive clarity [16]. By 1996, the first in vivo OCT scan of a human coronary artery was performed by Tearney et al. [17], made possible by the development of flexible instruments like catheters and endoscopes.

One of the main drawbacks of early OCT technology—specifically time-domain OCT (TD-OCT)—was its slow image acquisition speed and the necessity to temporarily displace blood, which interfered with image clarity due to its high scattering properties. These limitations were largely overcome with the advent of optical frequency domain imaging (OFDI), a faster and more sensitive second-generation OCT technology that has since become the standard in cardiovascular imaging [3].

Today, OCT has evolved into a critically important tool for both the diagnosis and treatment guidance of CAD. Its ability to provide real-time, high-resolution imaging of vascular structures allows for precise identification of atherosclerotic plaque characteristics and supports accurate diagnosis as well as treatment guidance. This detailed visualization supports more precise, tailored therapeutic decisions, enhancing both safety and efficacy in PCI. As a result, OCT is now considered an essential component of modern interventional cardiology, playing a central role in delivering personalized care.

Although OCT offers unmatched spatial resolution and unique capabilities in assessing plaque morphology, thrombus composition, and stent optimization, it is important to acknowledge its limitations and to contextualize its role among other intravascular imaging (IVI) modalities. One of its principal drawbacks is the limited tissue penetration—approximately 1 to 2 mm—which may hinder the evaluation of deeper vessel wall layers, especially in the presence of large thrombi or dense calcifications [3]. Moreover, OCT image acquisition requires transient clearance of blood using contrast media, which may be suboptimal or contraindicated in patients with renal dysfunction or hemodynamic instability [3,18]. The interpretation of OCT images is also operator-dependent, requiring specific training and experience, and the cost and limited availability of OCT systems may restrict its routine use in non-tertiary or resource-limited settings. Even in tertiary care centers where OCT technology is available, its adoption remains suboptimal due to high procedural costs and the steep learning curve associated with its interpretation. Given that OCT is a highly operator-sensitive modality, clinicians who perform it infrequently may lack the expertise needed to fully exploit its diagnostic potential, which can lead to suboptimal image acquisition or misinterpretation. In this context, the integration of artificial intelligence (AI) tools for real-time image analysis and interpretation represents a promising frontier. AI-assisted platforms could standardize OCT readings, reduce inter-operator variability, and support less experienced users in recognizing key pathological features, ultimately promoting broader, more effective use of this technology in clinical practice.

In comparison, IVUS, although characterized by lower spatial resolution (~100 µm), provides greater tissue penetration (up to 8 mm), allowing for the assessment of plaque burden, remodeling, and the external elastic membrane—particularly useful in large-caliber or ostial vessels, as well as in left main disease. IVUS is less affected by blood artifact and does not require contrast administration, making it more suitable in certain patient populations [3,19]. While OCT provides superior delineation of surface calcium and can measure calcium thickness with precision, IVUS is generally more informative in visualizing deep calcific structures and guiding lesion preparation in heavily calcified segments [3,18,19]. Near-Infrared Spectroscopy (NIRS), often integrated with IVUS, adds further biological insight by detecting lipid-rich plaques through spectroscopic analysis, offering a quantitative assessment of lipid burden along extended vessel segments. Unlike OCT and IVUS, however, NIRS does not provide structural imaging and lacks spatial resolution, thus serving primarily as a tool for compositional profiling and risk stratification [20,21]. Each of these imaging modalities, therefore, offers specific strengths and limitations, and their clinical application should be individualized based on the diagnostic goal, anatomical features, and patient context.

From a clinical perspective, OCT has gained increasing importance not only as a diagnostic tool but also as a procedural adjunct to optimize PCI. According to the latest ESC guidelines, the use of IVI—specifically OCT and IVUS—is recommended in several clinical settings. In patients with chronic coronary syndrome (CCS), IVI is recommended with Class I, Level A evidence to guide PCI in anatomically complex lesions, including left main (LM) disease, true bifurcations, and long lesions [22]. In such cases, OCT provides high-resolution visualization that supports precise lesion assessment, appropriate stent sizing, and post-PCI optimization, ultimately reducing the risk of stent failure and other adverse events. In ACS, the guidelines recommend IVI with Class IIa indication when there is a clearly identifiable culprit lesion on angiography, to optimize procedural strategy and stent deployment. Furthermore, OCT is specifically suggested with a Class IIb indication in cases of ambiguous culprit lesions, where standard angiography does not sufficiently clarify the underlying mechanism [23]. In particular, OCT proves especially useful in cases falling under the definition of MINOCA, where it enables the identification of non-obstructive PR or PE, coronary vasospasm, SCAD, or coronary embolism—mechanisms that are often angiographically silent or ambiguous but clinically significant (Table 1).

## 3. Myocardial Ischemic Syndromes: A New Nomenclature

For decades, ischemic heart disease has been predominantly attributed to atherosclerotic obstructions in the epicardial coronary arteries, shaping both diagnostic approaches and treatment strategies. However, recent advancements in cardiovascular research have highlighted the role of non-obstructive mechanisms, demonstrating that myocardial ischemia can occur even in the absence of significant coronary artery obstruction [24]. This evolving perspective emphasizes the central role of pathophysiology in ischemic heart disease, recognizing that coronary artery stenosis alone does not fully account for the complexity of myocardial ischemia with emerging evidence highlighting the significance of non-obstructive causes, such as epicardial coronary spasm, coronary microvascular dysfunction (CMVD), MB, and extramural microcirculatory compression, in contributing to ischemic events [22,23]. To better reflect this pathophysiological diversity, a new classification system has been proposed, distinguishing between AMIS and NAMIS, which better integrates both epicardial and non-obstructive ischemic conditions, moving away from the traditional “stenosis-centered” model. The proposed binary classification introduces “acute” and “non-acute” myocardial ischemic syndromes, refining the existing terminology to include a broader range of ischemic conditions [2]. Within AMIS, a subdivision is established for ACS, alongside another category encompassing all causes of MINOCA. Similarly, NAMIS includes patients with non-acute obstructive coronary disease, distinguishing them from those experiencing INOCA [2]. This framework ensures that both epicardial and microvascular causes of ischemia are recognized, promoting a more precise and individualized approach to patient care. In this evolving scenario, a paradigm shift in diagnosing and managing ischemic heart disease is encouraged through the integration of advanced intravascular imaging techniques, such as IVUS, OCT, and coronary functional tests (CFT).

Thus, the understanding of myocardial ischemia has advanced significantly, necessitating a more refined and inclusive classification system. By transitioning from an “anatomy-based” model to a “pathophysiology-centered” approach, clinicians can achieve greater diagnostic accuracy, tailor treatment strategies to individual patients, and ultimately improve clinical outcomes. However, the successful adoption of this framework will require broad consensus among international cardiovascular societies, integration into clinical guidelines, and continued research to optimize its practical application in cardiovascular medicine.

Precisely because “*it is in words and language that things first come into being and are*” [25], this new nomenclature represents more than a linguistic change—it marks a pivotal step in redefining our understanding and management of ischemic heart disease. By reshaping the terms we use, we reshape the reality we engage with, fostering clearer diagnosis, more precise treatment, and ultimately, better patient care. In this way, language becomes not just a tool for description, but a means of transformation.

## 4. The Role of OCT in Acute Myocardial Ischemic Syndromes

### 4.1. Exploring the Pathophysiology of Acute Coronary Syndromes: The Pivotal Role of OCT

While angiography continues to be the most widely used technique for detecting culprit lesions in ACS, its limitations as a two-dimensional imaging technique become evident in cases with ambiguous angiography findings, multiple potential culprit sites, or when a clear culprit cannot be determined. In such scenarios, OCT provides a decisive advantage, delivering ultra-high-resolution, cross-sectional imaging of the infarct-related segment. Unlike traditional angiography, OCT enables precise visualization of plaque phenotype, thrombus composition and quantification, significantly enhancing diagnostic accuracy. More than just identifying lesions, OCT is essential for distinguishing the primary pathological processes responsible for intracoronary thrombosis: PR, PE, and CN [5,26,27].

Over the past few decades, clinical and pathological studies have significantly advanced the understanding of ACS pathogenesis. While atherosclerosis alone leads to luminal narrowing and stable angina, ACS is caused by thrombus formation over unstable plaques. PR is the predominant cause of ACS, but other mechanisms, such as PE and CNs have been identified [5,26,27].

#### 4.1.1. Plaque Rupture and “Plaque Vulnerability”

**Figure 1:**
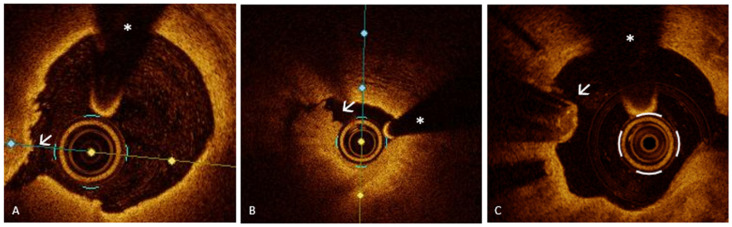
Mechanisms of Acute Coronary Syndromes. Panel (**A**): Plaque rupture: rupture of fibrous cap (white arrow) resulting in a large vessel wall cavity with exposure of highly thrombotic necrotic core to the blood flow without evidence of thrombi. Panel (**B**): Definite OCT-erosion: presence of thrombus overlying an intact plaque without evidence of fibrous cap rupture. Mixed thrombus at 6–11 ‘o clock (white arrow) with posterior shadowing with an underlying lipid-rich plaque. Panel (**C**): Eruptive CN with fibrous cap rupture and overlying mixed thrombus (white arrow) in the context of severely calcified in-stent restenosis. *The asterisk is indicating the guide wire artifact. All images come from the authors’ personal archive, unless otherwise indicated.*

PR represents the leading cause of ACS, responsible for approximately 60% of cases, followed in frequency by PE and, less commonly, CNs [26,27]. PR is characterized by a disruption of the fibrous cap, creating a cavity within the vessel wall (Figure 1, Panel A). This disruption exposes the thrombogenic necrotic core of the plaque to the bloodstream, triggering the formation of an intracoronary thrombus and the subsequent onset of ACS [26]. PR is the only ACS pathway with a well-defined precursor lesion: the thin-cap fibroatheroma (TCFA) [28,29,30]. TCFA is a plaque with a large lipid core (lipid arc ≥ 90°) covered by a thin fibrous cap (≤65 μm) [3,29], serving as a key marker of plaque vulnerability [29], as demonstrated by several studies that have linked this plaque phenotype to the occurrence of major adverse cardiac events (MACE) [20,21,31,32]. Notably, the presence of TCFA is not limited to culprit lesions but reflects widespread coronary vulnerability with non-culprit lesions often sharing similar high-risk features, predisposing patients to future acute ischemic events [33,34,35]. In this context, various trials have explored the hypothesis that preventive stenting of vulnerable plaques could be an effective strategy to reduce MACE. In the PROSPECT ABSORB trial [36], 898 patients with myocardial infarction (MI) were enrolled to evaluate the potential of pre-emptive intervention on high-risk plaques. Following the treatment of all flow-limiting lesions, IVUS was used to detect vulnerable plaques. Participants were then assigned to receive either standard medical therapy alone or in combination with a bioresorbable vascular scaffold (BVS). After a 25-month follow-up, although the BVS group demonstrated a larger minimal lumen area (MLA), this did not translate into a statistically significant reduction in MACE compared to medical therapy alone. The PECTUS trial [37], a similarly designed study, was prematurely terminated due to the market withdrawal of the Absorb BVS, preventing the collection of conclusive data. Finally, the PREVENT trial [38]—the largest study of its kind—included 1606 patients with both ACS and CCS and non-obstructive but high-risk plaques detected by OCT, IVUS, or NIRS. Results showed that preventive PCI led to a significant reduction in MACE compared to medical therapy alone, supporting early intervention when vulnerable plaques are identified through advanced imaging. However, despite these promising findings, significant limitations temper the clinical applicability of such strategies. The PREVENT trial, while being the largest and most comprehensive to date, presents several critical limitations that warrant cautious interpretation. Firstly, the study population was geographically limited, with enrollment restricted to South Korea, Japan, Taiwan, and New Zealand. This raises concerns about the generalizability of the findings to broader, more diverse populations. Additionally, women represented only 27% of the cohort, a notable underrepresentation that limits insights into sex-specific responses to preventive PCI. Another important limitation lies in the trial’s open-label design, which inherently carries a risk of bias, particularly in relation to soft end-points like clinically driven target-vessel revascularization, which was the major contributor to MACE rate reduction. Furthermore, the overall event rate in these trials was low, potentially affecting the statistical robustness of the results. Moreover, only 2% of patients were treated with Proprotein Convertase Subtilisin/Kexin type 9 inhibitors, despite the known benefits of intensive lipid-lowering therapy in reducing plaque vulnerability [39,40,41]. This low percentage may have influenced the outcomes, given the critical role of lipid management in atherosclerosis progression. From an imaging standpoint, 97% of patients were assessed with IVUS, primarily characterizing plaque vulnerability based on plaque burden and MLA. While IVUS is a valuable tool, it does not capture other critical features of vulnerability—such as fibrous cap thickness or lipid core size—that are better identified by more advanced imaging techniques like OCT or NIRS. This reliance on IVUS may have led to an incomplete assessment of real “plaque vulnerability”. Moreover, it is noteworthy that most of the benefit of preventive PCI was observed within the first two years. This temporal limitation underscores a fundamental challenge in managing coronary vulnerability: despite initial preventive PCI, new vulnerable plaques can develop elsewhere over time, perpetuating the risk of future events. This dynamic nature of atherosclerosis suggests that it is neither feasible nor effective to pursue an endless “race” against plaque vulnerability through serial preventive interventions. Additionally, the trial lacked data on the cost-effectiveness of a preventive PCI strategy. In a healthcare landscape where resource allocation is critical, understanding the economic implications of such an approach is essential. Without clear evidence of cost–benefit, widespread adoption remains questionable. Another notable omission is that the trial did not account for periprocedural myocardial infarctions in its outcomes. Given that such events can significantly impact patient prognosis, excluding them from the analysis may have led to an underestimation of the actual procedural risk associated with preventive PCI. In light of these limitations, it is clear that while preventive PCI shows potential, we are still far from integrating this strategy into routine clinical practice. The complexity of coronary vulnerability, coupled with the dynamic nature of plaque progression and the practical challenges of widespread implementation, highlights the need for further, more robust studies. These should address the gaps identified—particularly in terms of imaging accuracy, long-term benefit, cost-effectiveness, and comprehensive outcome assessment—before preventive PCI can be confidently recommended as a standard strategy for reducing ischemic events.

#### 4.1.2. Plaque Erosion

PE causes up to 40% of ACS [8] and is characterized by endothelial dysfunction or loss without disruption of the fibrous cap [18,42,43]. On OCT imaging, PE is identified as the presence of thrombus or an irregular luminal surface without evidence of cap rupture. However, due to the limited resolution of OCT in detecting denudation of the endothelial monolayer, PE can be diagnosed with OCT only after excluding PR—specifically by confirming the presence of an intact fibrous cap underlying the thrombus [3,4]. OCT allows for the classification of PE into two categories: definite and probable. The key difference lies in the underlying plaque characteristics. In “definite” PE, thrombus is clearly visualized over an intact fibrous cap within a lesion that displays atherosclerotic features such as lipid accumulation or calcium (Figure 1, Panel B). In contrast, when thrombus or luminal irregularities are seen in the setting of an otherwise normal-appearing vessel, with no clear signs of atherosclerotic plaque, the diagnosis is considered “probable” PE [3]. Unlike PR, which follows a well-established pathological sequence, PE is more heterogeneous in nature and can develop across different plaque morphologies, often involving those considered more “stable” [26,42,44]. In recent years, multiple studies have focused on the pathobiological differences between PR and PE in the context of ACS. Current evidence shows that PR typically originates from lipid-rich plaques with a thin fibrous cap, infiltrated by inflammatory cells—especially macrophages—and degraded by matrix metalloproteinases. This process leads to cap disruption, exposure of the necrotic core, and formation of a fibrin- and erythrocyte-rich thrombus. In contrast, PE occurs without cap rupture and involves endothelial denudation over plaques rich in smooth muscle cells and proteoglycans, often lacking a necrotic core. Thrombi are mainly platelet-rich. Mechanisms underlying PE include endothelial cell apoptosis, oxidative stress, disturbed flow, and activation of neutrophil extracellular traps and Toll-like receptor 2 [45]. These distinct biological pathways not only differentiate erosion from rupture morphologically and immunologically but also suggest the need for tailored therapeutic strategies based on the underlying mechanism of ACS. In line with this evidence, the EROSION study [46] and its 1-year follow-up [47] strongly support a mechanism-guided approach in ACS. In cases of OCT-confirmed PE—where the fibrous cap remains intact and the thrombus forms over a structurally stable plaque—intensive anti-thrombotic therapy without stenting proved to be both safe and effective. At 1 month, the majority showed significant thrombus reduction, and at 1 year, 92.5% remained free of major adverse cardiovascular events. This contrasts with PR, which typically requires stenting due to severe structural disruption and higher thrombotic risk. These findings emphasize that OCT can guide personalized therapy, avoiding unnecessary stents in erosion and improving outcomes through tailored intervention strategies.

#### 4.1.3. Eruptive Calcified Nodule

A heavily calcified nodular lesion that protrudes into the coronary lumen defines a CN. A CN is classified as non-eruptive when it presents without disruption of the fibrous cap and overlying thrombus. In contrast, eruptive CN is a rare but important cause of ACS, responsible for approximately 5% of cases. In this form, the CN breaches the fibrous cap, leading to thrombus formation [48]. The OCT-based diagnosis of this ACS mechanism can be particularly challenging. It requires the identification of a prominent, nodular calcific lesion with clear evidence of fibrous cap disruption and thrombus, typically occurring within the context of extensive calcific atherosclerosis. However, the underlying plaque phenotype may not be readily visible at the culprit site, as thrombus-related backshadowing can obscure the vessel wall. As a result, careful examination of the entire pullback is often required to identify the surrounding calcific burden and support the diagnosis of an eruptive CN. This diagnosis depends not only on focal imaging features but also on a comprehensive assessment of the overall vessel disease phenotype (Figure 1, Panel C) [3,26,27].

### 4.2. OCT for Qualitative and Quantitative Assessment of Intracoronary Thrombosis

**Figure 2:**
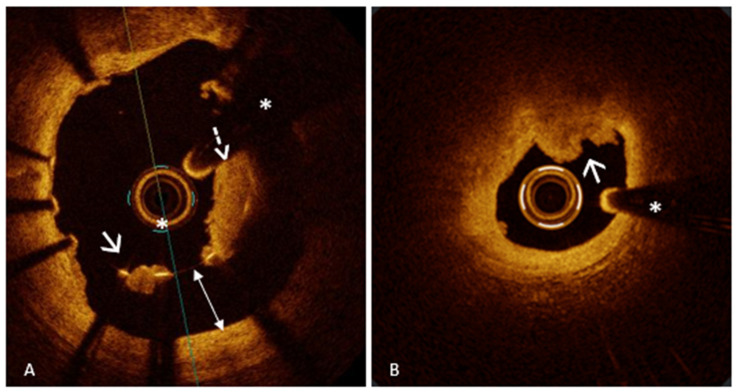
Thrombi. Panel (**A**): case of in-stent thrombosis in the context of major stent malapposition (the distance between the malapposed struts and the vessel wall is indicated by the double-headed arrow). A red thrombus is visible (high backscattering and posterior shadowing) with attenuation of the underlying plaque (dashed arrow), as well as a white thrombus (low backscattering and without posterior shadowing) (white arrow) Panel (**B**): mixed thrombus in the context of a lipid-rich plaque (definite OCT-erosion). *The asterisk is indicating the guide wire artifact. All images come from the authors’ personal archive, unless otherwise indicated.*

Traditional coronary angiography has significant limitations in the assessment of intracoronary thrombi as it provides only indirect visualization of thrombotic material. Advanced analytic methods such as dual quantitative coronary angiography (dual-QCA), which combines edge-detection and video-densitometry to estimate thrombus volume, have expanded angiography’s capabilities. However, even with dual-QCA, angiography remains an indirect modality with limited sensitivity and accuracy compared to direct IVI techniques [49]. In this scenario, OCT provides unparalleled insight into intracoronary thrombus, offering diagnostic information that no other imaging modality can match. Thanks to its high resolution and optical clarity, OCT allows for the precise identification of intraluminal thrombi as irregular masses, either adherent to the vessel wall or freely floating within the lumen [3]. Beyond simply identifying thrombus, OCT allows for differentiation between thrombus morphologies and compositions based on their optical properties. Red thrombi, which consist mainly of erythrocytes, typically appear on OCT as structures with high backscattering and significant signal attenuation, often accompanied by posterior shadowing (Figure 2, Panel A). In contrast, white thrombi, primarily composed of platelets, generate a lower-intensity signal with minimal posterior attenuation (Figure 2, Panel A). OCT is also capable of detecting mixed thrombi, which exhibit a combination of features from both red and white thrombi, resulting in an intermediate optical profile (Figure 2, Panel B) [3,4,18]. When evaluating the culprit lesion in ACS, it is important to recognize that intracoronary thrombi may not always be visible, due to natural thrombolysis or prior administration of antithrombotic or thrombolytic therapy. As a result, the absence of a detectable thrombus does not necessarily exclude its initial presence.

In addition to qualitative thrombus assessment, OCT enables a semi-quantitative characterization using different scoring systems. The Prati’s Thrombus Score is a quantitative OCT-based method used to evaluate intracoronary thrombus burden, as introduced in the COCTAIL (ClearwayRx System to reduce intracoronary thrombus in patients with acute coronary syndromes according to Optical Coherence Tomography after Abciximab Intracoronary Local infusion) trial [50]. This scoring system determines the thrombus extent by assessing the number of quadrants occupied by thrombus in each OCT cross-section during a pullback. Each cross-section is categorized as having no thrombus (score 0) or thrombus affecting 1, 2, 3, or all 4 quadrants (scores 1 to 4, respectively). The total thrombus burden is then calculated as the sum of scores across all cross-sections, providing a comprehensive measure of the thrombotic load. In addition to scoring, OCT allows for thrombus volume quantification [51]. This is performed by tracing the thrombus area across multiple frames, calculating the average area, and multiplying it by the thrombus length. This method provides a more precise measurement of thrombotic burden, helping to tailor patient-specific treatment strategies [52].

In conclusion, OCT has emerged as the gold standard for detecting intracoronary thrombi, particularly in cases where angiographic imaging is inconclusive or ambiguous. Due to its superior high-resolution imaging capabilities, OCT can provide critical clarity in identifying thrombi that may otherwise be overlooked with conventional angiography, allowing for a more precise assessment of culprit lesions, playing a pivotal role in optimizing interventional strategies and improving patient outcomes in ACS management.

### 4.3. Stent Thrombosis as a Cause of ACS: The Role of OCT in Detection and Understanding Underlying Mechanisms of Early and Late Stent Failure

Despite substantial progress in drug-eluting stent (DES) technology, PCI techniques, and antithrombotic regimens, stent thrombosis (ST) continues to pose a significant risk due to its potential to cause sudden cardiac events and accounts for approximately 20% of post-procedural MI [53]. The Academic Research Consortium (ARC) system defines ST based on diagnostic confidence—categorizing events as definite, probable, or possible—and according to the timing of occurrence: early (within 30 days), late (31 days to 1 year), and very late (beyond 1 year) [54]. The occurrence of ST results from a multifactorial interplay involving patient-specific, lesion-related, and procedural components. From a clinical perspective, individual susceptibility is heightened in the presence of comorbidities such as diabetes mellitus, chronic renal impairment, malignancies, peripheral vascular disease, and younger age [55]. Premature withdrawal of dual antiplatelet therapy (DAPT), ACS as the initial presentation, hereditary factors, and compromised left ventricular function [56,57] also increase vulnerability to early thrombotic events.

Lesion-related contributors include LM involvement, small vessel disease, heavy thrombotic burden, bifurcation lesions, and extensive calcification. Stent-related risks depend on the timeline of ST, with thin struts and small diameters increasing the likelihood of early ST, multiple overlapping stents predisposing to later events, and excessive stent length playing a role in both. Procedural factors further contribute to ST development with inadequate stent expansion and apposition, stent edge dissection and “geographical miss” being particularly critical for early ST. In contrast, delayed healing processes, exposure of metallic struts to circulating blood elements, acquired late malapposition, overlapping stents, and progressive neointimal atherosclerosis have been associated with late and very late events [53,55,56,57]. Given the multifactorial nature of ST, OCT has become an invaluable tool in both prevention and diagnosis, enabling real-time detection of lesion and procedural risk factors and precise characterization of failure mechanisms to guide individualized treatment.

OCT is an invaluable tool for assessing the mechanisms of stent failure underlying ST, including underexpansion, malapposition, and stent edge dissection (SED), all of which significantly impact post-PCI outcomes. Stent underexpansion, in particular, is a well-established predictor of adverse events, as insufficient expansion can lead to impaired blood flow and increased thrombotic risk. Among the key parameters in evaluating PCI success, minimum stent area (MSA) plays a fundamental role. The DOCTORS trial [58] highlighted the strong correlation between suboptimal MSA and a higher incidence of major cardiovascular events, while findings from the CLI-OPCI registries identified 4.5 mm^2^ as a threshold for predicting major cardiovascular events [59]. This threshold is even higher for LM disease and current European Association of Percutaneous Cardiovascular Intervention (EAPCI) guidelines recommend MSA values exceeding 7 mm^2^ for distal LM and >8 mm^2^ for proximal LM to optimize clinical outcomes and prevent late adverse events [6]. More broadly, a relative stent expansion >80% is considered a key target for PCI optimization [6]. Reaching this level of expansion is essential not only for minimizing the risk of ST but also for preventing in-stent restenosis and impaired vessel healing. By providing high-resolution, real-time imaging, OCT plays a fundamental role in ensuring optimal stent expansion, allowing for immediate identification and correction of underexpansion, thus enhancing PCI success and long-term patient outcomes.

Stent malapposition (SM), defined as a distance between stent struts and the vessel wall that exceeds struts thickness, is another key factor contributing to ST. Acute stent malapposition (ASM) arises at the time of stent deployment and is commonly attributed to stent undersizing relative to the actual vessel dimensions. Another frequent cause is the implantation of the stent across an ectatic segment, which can result in a localized area of malapposition at the site of the ectasia, despite optimal apposition of the stent edges to the vessel wall both proximally and distally [60,61]. In contrast, late SM (LSM) can either result from a persistent acute malapposition that fails to resolve (late persistent SM) or can develop progressively due to vascular remodeling (late acquired SM) [62]. While ASM in approximately half of cases self-corrects [60], LSM has been linked to a higher risk of adverse events, with this risk increasing proportionally to the total malapposition volume (TMV) [63]. According to EAPCI guidelines, ASM should be corrected if it exceeds 0.4 mm for >1 mm in length, as smaller areas are more likely to resolve naturally over time [6]. In this context, OCT plays an irreplaceable role in the prevention, diagnosis, and treatment of SM. It helps prevent malapposition through accurate stent sizing and proper lesion preparation. Beyond prevention, OCT enables the detection of both ASM and LSM, offering precise quantification by measuring the distance between struts and the vessel wall, assessing strut thickness, and evaluating the longitudinal extension of malapposition. This level of detail allows interventional cardiologists to determine whether correction is necessary and assess the effectiveness of post-dilation, ensuring optimal stent apposition and minimizing the risk of future complications.

Another major contributor to ST is SED, which OCT detects with far greater sensitivity than IVUS. According to the ILUMIEN III study [64], SEDs are categorized as major or minor, with major dissections defined as those extending ≥60° circumferentially or >2 mm in length. In this scenario, OCT proves to be an essential tool, as it enables the prevention of SED through precise lesion preparation, optimal stent placement, and controlled post-dilation, all tailored to the morphology and length of the plaque. Additionally, OCT allows for real-time detection of SED immediately after stent deployment, facilitating prompt intervention to correct the dissection and thereby reducing the risk of ST and in-stent restenosis, ultimately improving long-term outcomes.

Of note, another risk factor for late and very late ST is the presence of uncovered stent struts, as highlighted by data from the PRESTIGE [65] and PESTO [66] registries. Although many stent struts could remain exposed shortly after implantation, biological mechanisms—including neointimal formation and endothelial cell regrowth—typically lead to progressive coverage of the struts during the healing process. According to OCT criteria, strut coverage is confirmed when tissue >0 µm is detected over a stent strut [67]. By identifying the number of uncovered struts and dividing it by the total number of analyzable struts, OCT also allows for the calculation of the uncovered strut [4]. However, persistently uncovered struts considerably increase the risk of late ST, particularly in patients with inadequate antiplatelet therapy [6,68].

Thus, in the specific setting of ST caused by stent underexpansion, SM, SED, and uncovered stent struts, OCT proves to be an invaluable tool by accurately identifying the underlying mechanism of stent failure responsible for ACS. Its high-resolution imaging allows for precise detection of these high-risk features, enabling targeted intervention to correct them effectively. By guiding appropriate therapeutic strategies, OCT significantly reduces the risk of recurrent cardiovascular events, optimizing both acute treatment and long-term patient outcomes.

It is important to emphasize that in the era of “modern PCI”, we should overcome the limitations imposed by coronary angiography, which has inherent constraints due to its two-dimensional nature. Especially in complex coronary anatomy, we should rely on the additional role of IVI using IVUS or OCT for the diagnosis and treatment of CAD. In this scenario, several randomized controlled clinical trials [69,70,71,72] have confirmed the superiority of IVI-guided PCI over angiography-guided PCI in terms of clinical and procedural outcomes. A recent meta-analysis has definitively crystallized this evidence. The meta-analysis demonstrated that IVI-guided PCI reduces the risk of MACE compared to angiography-guided PCI [73]. In this context, the OCTIVUS trial [74], a non-inferiority study, evaluated OCT versus IVUS guidance in PCI by comparing rates of cardiac death, target vessel MI, and target vessel revascularization at one year. With over 1000 patients in each group, the study confirmed that OCT was non-inferior to IVUS for the primary composite endpoint. A subsequent prespecified substudy [70] focused on complex lesions—including unprotected LM disease, bifurcations, aorto-ostial lesions, CTOs, severe calcification, long lesions, in-stent restenosis, and multivessel disease. At a median two-year follow-up, outcomes for OCT- and IVUS-guided PCI were comparable regarding major adverse cardiac events. However, OCT guidance was associated with fewer procedural complications and a lower incidence of target vessel MI.

Especially in the context of complex coronary lesions, IVI plays a pivotal role, providing essential information during each phase of the procedure. Prior to PCI, it is a diagnostic tool that offers valuable insights into the lesion’s composition and characteristics, identifies possible mechanisms of stent failure in cases of in-stent restenosis, and precisely determines lesion length and vessel lumen area, guiding the correct sizing and positioning of the stent. During PCI, it serves as a guide for lesion preparation and confirms the effectiveness of the procedure. Finally, after stent placement, it allows for detailed evaluation, detecting potential complications such as malapposition, underexpansion, deformation, or dissections [75].

### 4.4. The Role of OCT in Myocardial Infarction with Non-Obstructive Coronary Arteries

MINOCA is a clinical condition characterized by AMI without significant obstruction in the epicardial coronary arteries (less than 50% stenosis) as detected by coronary angiography and no obvious non-coronary causes of type 2 AMI [76]. Its prevalence among AMI patients ranges from 3% to 15%, with a higher occurrence in women compared to men [77]. Type 1 MINOCA is caused by disruption of non-flow limiting atherosclerotic plaques while type 2 MINOCA can be caused by coronary embolism, coronary or microvascular spasm and SCAD [24]. Several recent observations suggest that Takotsubo syndrome is an additional cause of MINOCA as the key pathogenetic mechanism is microvascular spasm. Diagnosing MINOCA presents challenges due to its different causes. Coronary angiography, along with advanced IVI techniques such as IVUS and OCT, play an important role in the diagnostic process. Given the diverse nature of MINOCA, multiple additional imaging modalities are often required, including resting echocardiography, cardiac magnetic resonance (CMR), IVI techniques, and functional tests for myocardial ischemia, to accurately determine the underlying cause [23,78].

#### 4.4.1. OCT Findings in Type 1 MINOCA

Type 1 MINOCA results from the same atherosclerotic mechanisms that cause AMI with obstructive coronary disease, specifically PR, PE, and CNs. However, in contrast to “obstructive” AMI, Type 1 MINOCA does not present with a significant stenosis (>50%) in the epicardial coronary arteries on angiography, making it challenging to identify a clear culprit lesion. This scenario poses a diagnostic challenge in the catheterization laboratory and underscores the importance of intravascular imaging techniques to detect atherosclerotic causes that may otherwise remain undiagnosed.

High-resolution imaging modalities such as OCT and IVUS are essential in these cases. In Type 1 MINOCA, OCT findings—PR, PE, and CNs—mirror those observed in classic AMI, but with the critical difference that the stenosis is not obstructive. OCT can reveal subclinical ruptures, erosions, or CNs that, at the time of angiography, may not result in visible thrombosis, may have caused a transient thrombotic event that has since resolved, or may have led to distal embolization that remains undetected on standard angiographic imaging. IVUS and OCT studies have demonstrated that PR frequently occurs in MINOCA, with approximately one-third of patients showing evidence of this phenomenon [79]. OCT is regarded as the preferred imaging modality for its detection, as it provides high-resolution visualization of plaque disruption that may not be apparent on conventional coronary angiography. Other potential mechanisms in MINOCA include PE, characterized by the presence of a thrombus over an intact fibrous cap, and CNs, which appear as low-signal, irregular structures with blurred edges protruding into the arterial lumen. Although they are more frequently observed in obstructive AMI, they could also be potential mechanisms underlying MINOCA [24].

Since atherosclerotic lesions in MINOCA do not lead to significant epicardial obstruction, IVI is crucial for identifying the underlying pathology. Detecting these mechanisms is essential for guiding appropriate management strategies and improving secondary prevention in patients with MINOCA [23].

In this setting, although OCT remains the gold standard for elucidating the underlying mechanisms of type 1 MINOCA—owing to its unmatched spatial resolution and capacity to directly visualize fibrous cap integrity and intracoronary thrombus—IVUS also holds a valuable complementary role, particularly when OCT is not available or contraindicated. Thanks to its greater tissue penetration depth (4–8 mm) and independence from contrast agents, IVUS is especially advantageous in patients with renal impairment, hemodynamic instability, or in centers where OCT is not routinely accessible [7,24]. While IVUS cannot resolve microstructural features such as thin-cap fibroatheromas or subtle erosions, it enables detailed assessment of deeper plaque architecture, vessel remodeling, and calcific burden, which are often beyond the imaging capabilities of OCT [3]. In this context, the integration of NIRS with IVUS further enhances its diagnostic value by offering insight into plaque composition—particularly the presence of lipid-rich cores, a hallmark of vulnerable plaques. A compelling study by Terada et al. [80] investigated the utility of NIRS-IVUS in differentiating between the key substrates of acute coronary syndromes: PR, PE, and CN. According to their findings, PR typically appears on IVUS as an echolucent cavity within the plaque, often with superimposed thrombus, positive remodeling, and high plaque burden. These lesions also exhibit elevated lipid core burden index (LCBI) values on NIRS, reflecting their lipid-rich nature. Conversely, PE tends to present with an intact lumen contour and no visible rupture cavity on IVUS. These plaques are associated with lower LCBI values on NIRS and minimal or absent positive remodeling, indicating a more fibrotic and stable phenotype. Such morphological and compositional patterns provide indirect but useful clues that can help distinguish PE from PR when OCT is not feasible. In conclusion, while OCT remains the imaging modality of choice for diagnosing subtle endoluminal alterations in MINOCA, IVUS—particularly when combined with NIRS—can serve as a practical and informative alternative for the detection of PR and PE. In this regard, it may represent a valuable tool in the diagnostic workup of type 1 MINOCA, where non-flow-limiting plaque disruptions—such as ruptures and erosions—are among the leading underlying causes. By offering complementary morphological and compositional insights, especially in scenarios where OCT is not feasible or available, the IVUS-NIRS approach broadens the armamentarium for identifying the pathophysiological substrates of non-obstructive myocardial ischemia.

#### 4.4.2. OCT Findings in Type 2 MINOCA

##### Spontaneous Coronary Artery Dissection

SCAD is a cardiovascular disorder defined by a spontaneous, non-iatrogenic splitting of the coronary artery wall layers. This disruption permits blood to collect within the vessel wall, leading to the creation of an intramural hematoma. As the hematoma expands, it compresses the true lumen (TL) of the artery, reducing or completely obstructing blood flow [81]. This reduced blood supply can lead to various clinical scenarios, potentially culminating in AMIS. While the exact etiology of SCAD remains unclear, multiple predisposing factors have been recognized, such as vigorous physical activity, pregnancy or the postpartum period, fibromuscular dysplasia, and smoking habit [82]. SCAD is thought to develop through two main mechanisms: the “inside-out” model, where a tear in the intima allows blood to enter the media forming a false lumen (FL) that compresses the TL, and the “outside-in” model, where bleeding from small vessels within the media creates an intramural hematoma, narrowing the TL and mimicking atherosclerotic disease on angiography [82]. SCAD can be divided into four categories based on their angiographic appearance. Type 1 is marked by the visible passage of contrast dye along the artery wall, often accompanied by the detection of multiple luminal channels, and represents approximately 29–48% of cases. Type 2, the most prevalent form, observed in 60–75% of patients, presents as an extended, uniform narrowing of the vessel, which can be classified depending on whether the dissection reaches the distal end of the artery. Type 3, seen in about 3% of cases, manifests as a short, focal, and indistinct narrowing that can easily be mistaken for an atherosclerotic plaque. Finally, Type 4 involves a complete occlusion, typically affecting the distal segments of the coronary arteries [81,82,83]. In situations where conventional coronary angiography may be insufficient to accurately diagnose certain types of SCAD—particularly types 2, 3, and 4—IVI techniques play a pivotal role. Among these, OCT stands out as one of the most advanced and effective diagnostic tools [4]. This IVI technique enables detailed visualization of key diagnostic features of SCAD such as intimal tears, dissection flaps, and intramural hematomas. Its high-resolution imaging capability allows for accurate differentiation between the true and false lumens and facilitates the evaluation of the extent and severity of the intramural hematoma [3,4,84]. One of the hallmark OCT features of SCAD is the identification of a “flap” within a normal “three-layered” vessel segment. In addition, OCT is highly sensitive in identifying intramural hematoma, which may form through two previously mentioned mechanisms [3,84]. It is crucial to recognize that although identifying an intimal flap definitively confirms the diagnosis of SCAD, its absence does not rule out the condition. In fact, the mere presence of an intramural hematoma, even without a visible intimal tear, holds substantial diagnostic value. Accumulation of blood within the vessel wall, as seen in an intramural hematoma, can independently establish the diagnosis, highlighting the need for careful imaging interpretation and a high index of suspicion when evaluating patients suspected of having SCAD. An interesting study conducted by Jackson et al. [84] provided important insights into the classification and pathophysiology of SCAD, distinguishing between “fenestrated” and “non-fenestrated” SCAD using OCT. In fenestrated SCAD, there is a communication between the TL and the FL, allowing contrast media to pass through and partially equalize pressure. Conversely, non-fenestrated SCAD lacks this connection, resulting in higher intramural pressure within the FL, greater external elastic lamina expansion, and more severe compression of the TL. The study found that non-fenestrated dissections showed larger FL areas and greater external elastic lamina (EEL) expansion, supporting the hypothesis that higher FL pressure leads to more severe luminal narrowing. Over time, EEL reduction indicated vessel remodeling as part of the healing process. Additionally, the study explored the role of the vasa vasorum (VV), small vessels that supply the arterial wall, hypothesizing that their rupture might contribute to SCAD initiation. However, no significant differences in VV density were observed between SCAD patients and controls. Interestingly, OCT identified microvessels traversing the FL in some cases, suggesting that these may play a role in the progression or healing of the dissection. Finally, the study analyzed the light attenuation properties of the FL contents, finding them to be heterogeneous but with lower attenuation compared to whole blood or thrombus. This suggests that the FL may contain organized thrombus or other evolving material, reflecting different stages of hematoma maturation. This elegant research significantly enhances the understanding of SCAD pathophysiology and highlights the crucial role of OCT in distinguishing between its subtypes and informing clinical management.

It is important to remember that, despite its crucial role in diagnosing SCAD, OCT carries certain risks. The technique requires the injection of contrast media to displace blood and obtain clear images. In the context of SCAD, this contrast injection can be hazardous, potentially exacerbating the dissection, propagating the hematoma, and even leading to vessel closure. Additionally, the manipulation of imaging catheters within a fragile, dissected vessel increases the risk of procedural complications. Given these risks, OCT should be used selectively and judiciously. Its application is particularly valuable when clinical suspicion for SCAD is high, but angiographic findings are inconclusive or ambiguous.

In this context, IVUS emerges as a valuable alternative or complementary imaging tool. With deeper tissue penetration (up to 8 mm) and no need for contrast, IVUS allows a more comprehensive assessment of vessel architecture, including false lumen size, hematoma extent, and wall integrity [85]. IVUS may be particularly advantageous in high-risk anatomical settings or in cases where OCT is contraindicated. High-definition IVUS can assist in identifying true lumen location, guidewire placement, and planning PCI, especially in cases of total vessel occlusion (SCAD type 4) or ambiguous lesions (type 3), where discrimination from atherosclerotic plaques is essential. Nonetheless, IVUS has inherent limitations: its lower spatial resolution (approximately 100–150 µm) can hinder the distinction between homogeneous intramural hematomas and lipid-rich atherosclerotic plaques, particularly when used by less experienced operators. Moreover, its grayscale imaging may miss subtle disruptions in the intimal layer that would be readily detected by OCT [86].

In conclusion, while OCT offers unparalleled diagnostic precision in the evaluation of SCAD, its application should be judiciously considered due to the possible procedural risks involved. The decision to employ OCT should be guided by the complexity of the angiographic findings, the level of clinical suspicion, and a thorough assessment of the patient’s overall condition. When utilized appropriately, OCT can significantly enhance diagnostic accuracy, facilitate targeted treatment decisions, and ultimately improve clinical outcomes in patients with SCAD.

##### Epicardial Coronary Spasm

**Figure 3:**
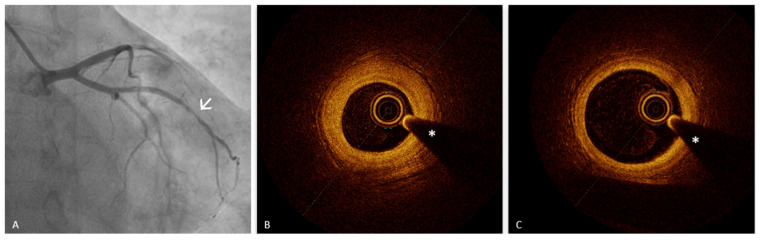
Epicardial spasm. Panel (**A**): coronary angiography showing focal coronary artery spasm after intracoronary acetylcholine administration, with >90% luminal diameter reduction; Panel (**B**): OCT pullback showing evidence of “short-in-length” luminal area reduction in a segment with intimal thickening; Panel (**C**): resolution of the spasm after intracoronary nitrate administration. *The asterisk is indicating the guide wire artifact. These images are courtesy of Francesco Fracassi, MD, PhD, with permission for use.*

Epicardial coronary spasm is defined as a transient constriction of an epicardial coronary artery, leading to a temporary reduction or interruption of coronary blood flow. This condition, characterized by a narrowing of more than 90% of the arterial lumen, typically presents with resting angina and is often accompanied by ischemic changes on the electrocardiogram (ECG) [24]. The spasm can occur unpredictably, either spontaneously or in response to external stimuli, and may be localized to a single coronary segment (focal) or extend to involve multiple segments (diffuse). The gold standard for diagnosing coronary vasospasm is the intracoronary acetylcholine (Ach) provocation test [87]. In this procedure, Ach is administered directly into the coronary artery to provoke a spasm. A positive diagnosis is established if the test induces a ≥90% constriction of the epicardial artery, alongside the reproduction of chest pain and ischemic ECG changes. The administration of intracoronary nitroglycerin is used to reverse the spasm, further confirming the diagnosis. This test is essential for detecting both focal and diffuse forms of vasospasm and is instrumental in guiding appropriate therapeutic decisions [87]. While angiography remains the primary diagnostic method to assess epicardial coronary spasm, OCT has proven to be an important adjunctive technique, offering additional diagnostic insights. OCT provides high-resolution imaging that allows for the detailed assessment of the structural characteristics of the coronary artery wall, revealing features that may not be visible on angiography [11]. In patients with vasospastic angina (VSA), OCT has demonstrated that the arterial segments involved in spasm often show diffuse intimal thickening, even in the absence of significant lipid or calcium deposits. This thickening can persist even when the artery is at rest, indicating a predisposition to vasospasm. During Ach-provoked spasm, OCT reveals additional distinctive findings such as “intimal bumps” that protrude into the arterial lumen and “intimal gathering”, where the thickened intima appears to fold or cluster, resulting in further luminal narrowing. These conformational changes typically resolve after the administration of nitroglycerin, indicating the dynamic nature of the spasm (Figure 3) [11,88].

Importantly, OCT is capable of visualizing the VV, a microvascular network that supplies the walls of large arteries [3]. The proliferation of these vessels is believed to play a role in the development of coronary artery spasm [89]. By promoting local inflammation and enhancing the reactivity of vascular smooth muscle cells, the VV may facilitate the onset of vasospastic events. This is supported by findings from Nishimiya et al., who demonstrated that patients with VSA exhibit a significant increase in adventitial VV formation, which correlates with the severity of coronary vasoconstriction and Rho-kinase activity, a key mediator of smooth muscle contraction [89]. These insights underscore the value of OCT in detecting vascular changes that may predispose to or sustain vasospastic episodes, thereby enhancing diagnostic accuracy and informing targeted therapeutic strategies. In conclusion, while Ach provocation testing remains the cornerstone for diagnosing epicardial coronary spasm, OCT serves as an essential adjunct by providing high-resolution structural insights. The ability of OCT to detect intimal thickening, medial changes, and the presence of VV offers a more comprehensive understanding of the mechanisms driving coronary vasospasm. This not only enhances diagnostic accuracy but also aids in selecting the most appropriate treatment strategy.

##### Coronary Embolism

Coronary embolism is a relatively rare cause of AMIS, resulting from the occlusion of a coronary artery by embolic material originating from another location within the circulatory system [7,24]. Potential embolic sources include thrombi from the left atrium in patients with atrial fibrillation, vegetations from infective endocarditis, or debris from ruptured atherosclerotic plaques. The clinical manifestations of coronary embolism can span the entire spectrum of ischemic presentations, up to and including MI, depending on the size and location of the embolic obstruction [24]. Diagnosing coronary embolism can be particularly challenging through conventional coronary angiography, as embolic material may not present with distinct imaging features or may mimic other coronary obstructions, such as PR. OCT, with its superior resolution, could be a valuable tool for the detection and characterization of embolic material within the coronary arteries [3]. OCT can identify intraluminal masses that typically appear as floating, irregular structures without an underlying plaque [3]. These embolic materials can exhibit several optical characteristics depending on their origin—thrombi may appear as heterogeneous, low-signal masses, whereas calcific or tissue-derived emboli may have different reflective properties. However, it is important to highlight that the role of OCT in detecting coronary embolism remains somewhat dissertative. In this review, we aim to discuss the role of OCT across all causes of AMIS and NAMIS, including coronary embolism. However, it is worth noting that addressing the role of OCT in the context of embolism remains largely an exploratory discussion, as definitive evidence is still limited. Nonetheless, OCT can be particularly useful when the embolic material originates from a ruptured or eroded and thrombosed plaque, with distal embolization of thrombotic fragments. In such cases, according to how far the catheter can be advanced distally within the vessel, this imaging tool can provide crucial insights. It allows for the identification of distal thrombotic material and can help establish a connection to an upstream plaque destabilization within the same artery. This diagnostic capability is essential for differentiating embolic phenomena from isolated thrombotic events and can significantly guide clinical management by revealing the underlying pathophysiological mechanism. The real diagnostic challenge lies in differentiating embolic occlusions from other entities, particularly OCT-detected “probable PE”. In OCT imaging, a “probable” erosion is characterized by the presence of thrombus over a “normal” vessel, without atherosclerotic features [3,4,18]. The visual similarity between embolic material and thrombus associated with probable erosion can lead to diagnostic uncertainty. Differentiating between these two scenarios requires careful consideration of clinical context, including the presence of potential embolic sources (e.g., atrial fibrillation, endocarditis) and overall patient history. The diagnostic approach must integrate OCT findings with angiographic data, clinical suspicion, and the presence or absence of embolic risk factors. Ultimately, while OCT is an indispensable tool for identifying intraluminal masses, its interpretation in the context of coronary embolism requires a holistic diagnostic approach. The differentiation from PE, for instance, is not always straightforward and depends on both imaging characteristics and the broader clinical scenario. Recognizing the absence of an underlying plaque, understanding the patient’s embolic risk profile, and correlating with clinical findings are essential for accurate diagnosis and appropriate therapeutic decisions.

## 5. The Role of OCT in Non-Acute Myocardial Ischemic Syndromes

In the landscape of NAMIS, OCT has emerged as an invaluable imaging modality, offering unparalleled insights into the complex mechanisms underlying non acute ischemia. Specifically, OCT plays a pivotal role in the detailed assessment of obstructive epicardial coronary stenoses. Its ultra-high resolution allows for a precise evaluation of stenosis characteristics, both in quantitative and qualitative terms, enabling an in-depth assessment of plaque morphology, plaque vulnerability, and the dynamic process of plaque healing. In the specific context of INOCA, OCT can contribute significantly to the diagnostic process. When traditional angiography fails to detect subtle epicardial abnormalities that may underlie ischemic symptoms, OCT can provide crucial insights. In this review, we will explore the OCT features associated with epicardial coronary spasm and MB. Although OCT is not considered the first-line modality for diagnosing these specific conditions, it can still offer meaningful information that enhances the understanding of their morphological and functional implications.

### 5.1. OCT Assessment of Anatomic Severity of Epicardial Stenoses and Plaque Phenotype

While angiography continues to be the most widely utilized method for evaluating epicardial coronary stenoses, advancements in OCT have introduced innovative approaches to both anatomical and functional lesion assessment. OCT now enables precise quantification of stenosis severity from both anatomical and functional perspectives, guiding decisions on revascularization. Moreover, OCT offers unique insights into plaque morphology, which can further refine strategies for PCI.

OCT is instrumental in determining the need for revascularization in patients with CCS, especially when symptoms persist despite medical therapy [22]. While invasive functional assessments like Fractional Flow Reserve (FFR) and Instantaneous Wave-Free Ratio (IFR) are established standards for ischemia detection [87,90], OCT offers complementary anatomical insights. By precisely measuring the MLA, minimal lumen diameter (MLD), and calculating percentage area stenosis (%AS), OCT facilitates accurate lesion severity assessment [91]. Although current guidelines prioritize pressure-based indices, emerging evidence suggests that OCT-derived measurements can predict hemodynamic significance, assisting in revascularization decisions. However, variability in anatomical cut-offs based on lesion location and myocardial territory highlights the necessity for individualized interpretation. In the FORZA trial [92], 350 patients with 446 angiographically intermediate coronary lesions (AICLs) were randomized in a 1:1 ratio to either the FFR or OCT imaging arm. In the FFR arm, PCI was performed if the FFR was ≤0.80. In the OCT arm, PCI was indicated if the percentage area stenosis (%AS) was ≥75%, or if %AS was between 50–75% combined with a MLA < 2.5 mm^2^ or the presence of plaque rupture. The study concluded that OCT-guided PCI in patients with AICLs is associated with a lower rate of MACE or significant angina compared to FFR guidance. However, FFR guidance resulted in a higher proportion of patients managed conservatively and incurred in lower procedural costs. These findings highlight that while OCT may enhance clinical outcomes, FFR offers a more cost-effective strategy with fewer interventions. The 5-year follow-up results of the FORZA trial [93] provide important insights into the long-term outcomes of using OCT versus FFR in the management of AICLs. At 5 years, the rate of MACE was similar between the OCT-guided and FFR-guided groups. Of note, the OCT group had numerically lower rates of all-cause mortality, MI, and target vessel revascularization. Although earlier follow-up showed a lower rate of the composite outcome of MACE or significant angina in the OCT group compared to FFR, the long-term results suggest that both modalities provide comparable outcomes in terms of MACE. Notably, the OCT-guided approach led to higher procedural rates of PCI, with more stents implanted and larger stent diameters compared to FFR-guided procedures. Overall, the findings support the role of OCT as a valid alternative to FFR in guiding the management of AICLs. However, further large-scale trials are recommended to refine the understanding of specific clinical contexts where OCT or FFR may offer distinct advantages.

Beyond anatomical evaluation, OCT-derived data contribute to computational models estimating functional stenosis severity. Integrating OCT-based geometrical data with fluid dynamics has led to the development of indices like the Optical Flow Ratio (OFR). These computational assessments offer a rapid, less invasive method to gauge hemodynamic impact without inducing hyperemia. Although OFR shows strong correlation with FFR and promises high diagnostic accuracy, its clinical application remains limited. Additionally, these indices do not account for collateral circulation, and their influence on clinical outcomes compared to traditional measures is still under investigation [91]. Beyond its role in assessing anatomical severity and, through advanced applications, functional significance, OCT plays a pivotal role in defining the detailed morphology of epicardial coronary stenoses. OCT allows for an in-depth evaluation of plaque phenotype, offering valuable guidance for PCI. Moreover, by providing insights into plaque vulnerability and the dynamic process of plaque healing, OCT contributes to a deeper understanding of atherosclerosis—a chronic but inherently “dynamic” disease. The plaque phenotype assessing by OCT not only reflects the current state of the lesion but also narrates the natural history of the patient’s atherosclerotic disease. It may even offer predictive insights into how the disease is likely to evolve over time, enhancing personalized risk assessment and therapeutic planning.

**Figure 4:**
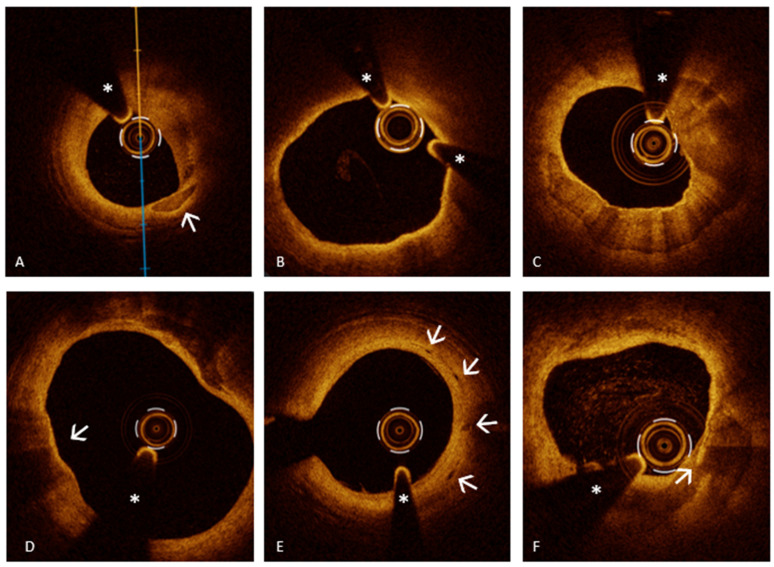
Plaque morphology and features. Panel (**A**): fibrous plaque with loss of typical three-layered structure characterized by a homogeneous region with high-intensity signal and an intimal thickness of ≥600 μm. “Spotty” calcium is indicated (white arrow). Panel (**B**): thin-cap fibroatheroma, lipid-rich plaque with a lipid arc >90° (signal-poor region with poorly defined borders) and a highly reflective thin fibrous cap (<65 μm). Panel (**C**): diffuse calcification, characterized by a region with low or heterogeneous intensity and sharply defined borders with an angular extension >90°. Panel (**D**): macrophages, recognized as confluent “bright spot” with posterior shadowing (white arrow). Panel (**E**): microvessel, recognized as small, round, low-intensity areas referred to as “small black holes” without contact with intimal layer (white arrows). Panel (**F**): cholesterol crystal, recognized as thin, linear, sharp-bordered regions with high intensity signal (white arrow). *The asterisk is indicating the guide wire artifact. All images come from the authors’ personal archive, unless otherwise indicated.*

OCT enables the distinction between a normal vessel, characterized by the typical trilaminar structure of the arterial wall—intima (high-intensity, “high backscattering,” homogeneous signal), media (low-intensity, “low backscattering” signal), and adventitia (high-intensity, “high backscattering” signal) [3,4,18]—and an atherosclerotic plaque, which is marked by the disruption of this normal trilaminar structure [3]. Atherosclerotic plaques can be further classified into several phenotypes based on the presence of lipids, calcium, and the thickness of the fibrous cap [94]. Fibrous plaques appear as homogeneous regions with high-intensity signals and an intimal thickness of ≥600 μm (Figure 4, Panel A) [3,4,18]. Calcification is identified as a region with low or heterogeneous intensity and sharply defined borders [3,4,18]. Calcium within atherosclerotic plaques may occur in both fibrous and lipid-rich regions, presenting either as focal “spotty” deposits or as extensive calcifications. OCT permits comprehensive characterization of calcific lesions, enabling precise quantification of their depth relative to the vessel wall, circumferential arc in degrees, and longitudinal extension along the vascular segment (Figure 4, Panel C) [95]. Lipid plaques are characterized as signal-poor regions with poorly defined borders and a highly reflective fibrous cap overlay [3]. When the angular extension of the lipid core exceeds 90°, the plaque is categorized as “lipid-rich” plaque or “fibroatheroma”. Fibroatheromas can be further classified based on the thickness of their fibrous cap (>65 μm or ≤65 μm) into two categories: “thick-cap” and “thin-cap” fibroatheromas [3]. TCFA, the prototype of a vulnerable plaque, is a lipid-rich plaque (lipid arc ≥90°) with a thin fibrous cap (≤65 μm) (Figure 4, Panel B) [3,29]. Moreover, OCT is the only IVI modality that allows for the visualization of vulnerability features such as macrophages, microvessels and cholesterol crystals (CCs). Macrophages appear as distinct or confluent high-intensity points, often described as “bright spots.” These bright reflections are typically accompanied by posterior shadowing due to the high optical attenuation of these cells (Figure 4, Panel D) [3,96]; microvessels represent the process of neoangiogenesis within the plaque. They are visualized on OCT as small, round, low-intensity areas referred to as “small black holes.” Importantly, these structures do not maintain continuity with the intimal layer of the vessel wall, differently from VV (Figure 4, Panel E) [97]; CCs are linear, highly reflective structures within the plaque, typically observed in the context of lipid-rich plaques (Figure 4, Panel F) [98,99,100].

In conclusion, OCT has emerged as a transformative tool in the comprehensive assessment of epicardial coronary stenoses. Its capacity to accurately evaluate both anatomical and functional lesion severity, guide revascularization strategies, and provide detailed morphological characterization of plaques offers significant clinical value. By identifying key features of plaque vulnerability and contributing to the understanding of dynamic processes such as plaque healing, OCT not only enhances immediate procedural outcomes but could inform long-term patient management. Furthermore, OCT’s detailed plaque phenotyping offers insights into the evolution of atherosclerotic disease, potentially enabling the prediction of disease progression and tailoring of personalized therapeutic strategies. As a result, OCT stands as a cornerstone in modern cardiovascular care, bridging the gap between diagnostic precision and personalized intervention, and ultimately contributing to improved clinical outcomes in patients with CAD.

### 5.2. OCT Findings in Ischemia with Non-Obstructive Coronary Arteries

INOCA is a complex and multifactorial condition characterized by the presence of myocardial ischemia without significant obstructive CAD on coronary angiography. Understanding the underlying causes of INOCA is essential for accurate diagnosis and effective management, as these patients face a heightened risk of MACE and frequently experience recurrent symptoms and hospitalizations [101,102]. INOCA is a common condition affecting an estimated 3 to 4 million individuals annually, with a marked female predominance. Approximately 50% of women undergoing coronary angiography show no significant obstructive CAD, compared to a smaller percentage in men [101].

One of the primary causes of INOCA is CMD, which involves impaired regulation of blood flow in the small coronary vessels. CMD can arise from structural abnormalities or dysregulation of vascular tone, leading to insufficient myocardial perfusion [101,102]. Specifically, CMD can provoke myocardial ischemia through two primary mechanisms. First, an excessive vasoconstrictive response in the microcirculation (i.e., microvascular spasm) can acutely reduce coronary blood flow, causing supply–demand mismatch and ischemia. Second, an inadequate vasodilatory reserve in the microvasculature—due to impaired vasodilation—leads to insufficient blood flow during increased myocardial demand. Notably, both mechanisms may arise from functional alterations that are either endothelium-dependent (e.g., reduced nitric oxide-mediated relaxation) or endothelium-independent (intrinsic hyperreactivity of vascular smooth muscle), reflecting the complex regulation of coronary tone [103]. In fact, epicardial coronary artery spasm, the hallmark of VSA, shares a similar pathophysiology: it results from a combination of endothelial dysfunction and heightened smooth muscle cell reactivity in the vessel wall [104]. Thus, whether the vasospasm occurs at the epicardial level or in the microcirculation, it is now understood as a conjoint effect of endothelial and vascular smooth muscle cells (VSMC) dysfunction rather than a purely endothelial or purely muscular phenomenon [105].

From a clinical perspective, these vasomotor dysfunctions underlie both NAMIS and AMIS, depending on their presentation. Patients with recurrent ischemic symptoms due to microvascular dysfunction or episodic epicardial spasm (as in microvascular angina or vasospastic angina)—without AMI—are categorized as having NAMIS [2]. These conditions represent stable forms of ischemic heart disease driven by microvascular or epicardial vasospasm. In contrast, an ACS can occur when severe coronary spasm or microvascular dysfunction precipitates myocardial injury despite no obstructive coronary arteries, a scenario classified as MINOCA [24,105,106]. Indeed, increasing evidence indicates that coronary spasm and related microvascular dysfunction are the precipitating cause in over half of MINOCA cases, while also being a major cause of chronic stable angina symptoms in patients with no obstructive disease [105]. In summary, CMD and coronary vasospasm lie at the intersection of NAMIS and AMIS, with endothelial and VSMC abnormalities jointly orchestrating both the non-acute and acute manifestations of ischemic heart disease.

MB, a congenital anomaly where a segment of the coronary artery tunnels through the myocardium, is also a notable cause of INOCA. This condition can lead to dynamic compression of the artery during systole, resulting in impaired coronary blood flow [107]. Rather than being a merely benign finding, MB can act as a “point of minor resistance,” predisposing to myocardial ischemia through multiple mechanisms. These include dynamic systolic compression of the artery, impaired diastolic perfusion, and functional abnormalities affecting both the endothelium and vascular smooth muscle cells [107]. Indeed, as highlighted by the RIALTO registry, MB-related ischemia may result from either epicardial or microvascular dysfunction, often involving a complex interplay of endothelial impairment and heightened smooth muscle reactivity. Full-physiology assessments have demonstrated that MB frequently coexists with coronary vasospasm and microvascular dysfunction, reinforcing the concept that MB is part of a broader spectrum of coronary circulatory disorders [108]. Additionally, diffuse atherosclerosis, even when non-obstructive, can contribute to INOCA by impairing blood flow without causing significant luminal narrowing detectable by angiography [109].

An interesting study by De Bruyne et al. demonstrated that non-stenotic coronary arteries in patients with CAD experience a gradual decline in pressure due to diffuse atherosclerosis. By measuring coronary pressure and FFR in both healthy individuals and patients with CAD, the study found that diseased arteries exhibited significantly larger pressure gradients and lower FFR values, indicating increased resistance to blood flow. In 57% of affected arteries, FFR was lower than in any healthy arteries, and in 8%, FFR fell below the ischemic threshold of 0.75. These findings suggest that diffuse atherosclerosis, even without focal stenosis, contributes to myocardial ischemia and has important implications for PCI [109]. In this scenario, advanced imaging techniques, such as OCT or IVUS, are critical in identifying these subtle forms of obstructive atherosclerosis that may be missed during routine angiography. The diagnosis of INOCA can be approached through both non-invasive and invasive techniques. Among non-invasive techniques, echocardiography, particularly transthoracic color Doppler, is used to evaluate CFR, defined as the ratio of peak hyperemic to resting coronary flow velocity. A CFR value of less than 2.5 is indicative of CMD. Myocardial contrast echocardiography also measures CFR, although it presents variability in image quality and reproducibility. Cardiac PET is one of the most reliable non-invasive techniques for evaluating INOCA. It assesses myocardial flow reserve (MFR), calculated as the ratio of MBF during pharmacological hyperemia to MBF at rest. An MFR greater than 2.3 is considered normal, whereas an MFR below 1.5 suggests CMD and an elevated risk of future cardiac events. CMR is another effective tool, utilizing the myocardial perfusion reserve (MPR) index to quantify MBF. An MPR index of 1.5 or less is considered abnormal, correlating with CMD. These non-invasive modalities collectively enhance the diagnosis and risk stratification for patients suspected of having INOCA. Invasive techniques involve coronary angiography followed by CFT. Scarsini et al. [87] have proposed a systematic algorithm, known as *#FullPhysiology*, for the comprehensive evaluation of patients with suspected CAD using advanced intracoronary physiological assessments. This method aims to enhance precision medicine by accurately identifying the pathophysiological mechanisms causing myocardial ischemia, thus facilitating tailored interventional or medical treatments. The *#FullPhysiology* approach systematically evaluates three primary aspects of coronary physiology. The first step involves the assessment of epicardial vessel function, beginning with non-hyperemic pressure ratios (NHPRs), such as the Resting Full-cycle Ratio (RFR), followed by the evaluation of FFR. Hyperemia is induced using pharmacological agents like adenosine to achieve accurate FFR measurements. A pressure wire pullback technique is also employed to create a detailed physiological map of the vessel, helping distinguish between focal, diffuse, or mixed disease patterns. This detailed mapping aids in understanding disease distribution and guides interventional strategies for better procedural planning.

The second step focuses on microcirculation assessment, recognizing that over 50% of patients presenting with anginal symptoms (ANOCA) or ischemia (INOCA) but without significant obstructive CAD may suffer from CMD. This assessment utilizes thermodilution-based techniques to measure CFR and the Index of Microcirculatory Resistance (IMR). A CFR ≤ 2.0 and an IMR ≥ 25 are indicative of CMD. The third step involves the evaluation of vasomotor function through vasoreactivity tests using Ach. This is crucial for detecting coronary vasospasm, whether at the microvascular or epicardial level. Epicardial vasospasm is diagnosed when there is a ≥90% constriction of the coronary artery, accompanied by typical chest pain and ischemic ECG changes. In contrast, microvascular spasm is diagnosed in the absence of significant epicardial constriction but with similar symptomatic and ECG presentations. These conditions are often transient and difficult to detect; thus, systematic testing is crucial for accurate diagnosis and management. Finally, post-PCI assessment is performed to confirm procedural success and ensure the long-term effectiveness of the intervention. Repeating the physiological assessment, particularly through FFR and pressure wire pullback, is essential to detect any residual focal lesions or diffuse disease. An optimal post-PCI FFR value is considered to be >0.90. Identifying and treating residual gradients can optimize outcomes and reduce the risk of post-procedural complications such as residual angina or target vessel failure.

The *#FullPhysiology* approach emphasizes personalized diagnosis and management by addressing all components of coronary physiology—epicardial disease, microvascular dysfunction, and vasomotor disorders. This systematic assessment ensures precise identification of the underlying cause of ischemia, leading to optimized patient outcomes, improved symptom management, and a reduction in adverse cardiovascular events. By integrating advanced diagnostic techniques within routine clinical practice, *#FullPhysiology* represents a significant advancement in the field of interventional cardiology. In this context, the routine use of OCT is not generally recommended, as the cornerstone of INOCA assessment, alongside angiography, is primarily based on coronary physiology. Nevertheless, OCT can offer valuable complementary information in selected cases, particularly in the presence of diffuse CAD. In such scenarios, OCT enables a more detailed quantitative and qualitative evaluation, which can be instrumental in refining the diagnosis and guiding subsequent treatment strategies. Furthermore, OCT can play a supportive role in assessing specific conditions like myocardial bridging and vasospasm, although it is not considered a first-line diagnostic tool for these disorders. In this review, we will delve deeper into the potential applications of OCT in these specific clinical contexts, emphasizing how its integration can enhance diagnostic accuracy and support more personalized therapeutic decisions.

#### Epicardial Coronary Spasm

Epicardial coronary vasospasm, while representing an unstable manifestation in MINOCA, constitutes the stable counterpart in patients with INOCA. Epicardial coronary vasospasm is characterized by a transient, intense constriction of the epicardial coronary arteries, leading to temporary myocardial ischemia [87]. Diagnosis relies on intracoronary ACh provocation testing, which identifies vasospasm through ≥90% arterial narrowing accompanied by angina and ischemic ECG changes [87,101,102]. Although ACh testing remains essential for functional diagnosis, OCT serves as a valuable adjunct by characterizing the structural substrate predisposing to vasospasm. As previously discussed, (Section Epicardial Coronary Spasm), OCT can reveal structural abnormalities such as diffuse intimal thickening, even in the absence of significant lipid or calcium deposits. During ACh-provoked spasm, OCT may demonstrate distinctive features like intimal bumps protruding into the arterial lumen or intimal gathering, where thickened intimal layers fold, contributing to further luminal narrowing. These changes are often reversible with intracoronary nitroglycerin, highlighting the dynamic nature of vasospasm. Additionally, OCT enables visualization of the VV, whose proliferation is associated with increased local inflammation and smooth muscle hyperreactivity, both of which are implicated in the pathogenesis of coronary spasm [89]. Thus, while OCT does not replace functional testing for the diagnosis of vasospasm, its ability to detect subtle structural abnormalities and vascular changes provides a deeper understanding of the mechanisms underlying vasospasm in INOCA. This enhances diagnostic precision and informs more targeted therapeutic strategies, ultimately contributing to improved patient outcomes.

## 6. Additional Information Provided by OCT Beyond AMIS and NAMIS

### 6.1. Plaque Vulnerability

The concept of plaque vulnerability—referring to an atherosclerotic lesion prone to destabilization and ultimately triggering ACS—has attracted substantial scientific attention over the past two decades. As our understanding of the pathophysiological mechanisms of plaque instability has evolved, IVI has played a pivotal role in elucidating both the phenotypic characteristics of high-risk plaques and the distinct mechanisms underlying ACS [26,27]. Among the available IVI modalities, OCT has emerged as the gold standard in this field, offering unparalleled spatial resolution that allows for the in vivo characterization of plaque morphology and the precise identification of the culprit mechanism behind ischemic events. Through OCT, clinicians and researchers can visualize with remarkable clarity features such as thin fibrous caps, large lipid-necrotic core, macrophage infiltration, CCs, microchannels—all features of plaque vulnerability [3,4,96,110,111]. IVI has not only deepened our comprehension of the biological underpinnings of ACS but also established clear links between specific vulnerable phenotypes and the risk of MACE [20,21,31,32]. Moreover, it has validated plaque burden as a powerful independent predictor of future events [20,33,34]. In essence, IVI imaging has demonstrated that both the qualitative nature (the type) and the quantitative extent (the burden) of atherosclerotic disease are key determinants of coronary vulnerability. Historically, TCFA has been regarded as the prototypical vulnerable plaque, most prone to rupture and responsible for a majority of ACS presentations [29]. However, a paradigm shift has emerged from a brilliant state-of-the-art review [8] recently published by leading experts in the field, led by R. Vergallo, which presents an updated and comprehensive redefinition of the concept of “vulnerable plaque”. This authoritative work synthesizes decades of research and advances in imaging to reshape our understanding of high-risk atherosclerotic lesions. Historically, vulnerable plaques were primarily identified as TCFAs, characterized by a large lipid-rich necrotic core covered by a thin fibrous cap. These plaques were considered the main precursors of PR, a predominant mechanism leading to ACS. However, recent evidence has shown that PE now accounts for up to 40% of ACS cases. This significant shift necessitates a broader definition of “high-risk plaques” that encompasses all major substrates contributing to coronary thrombosis, including rupture-prone plaques, erosion-prone plaques, and eruptive calcified nodules. Consequently, all precursors of the main mechanisms of ACS must be considered as “high-risk plaques”. In the pathophysiological landscape of high-risk plaque formation and instability, each substrate presents distinct mechanisms. Rupture-prone plaques are typically TCFAs, where the thin fibrous cap overlying a large lipid-rich core is susceptible to rupture, exposing the thrombogenic material to the bloodstream and triggering thrombosis [28,29,30]. In contrast, erosion-prone plaques are characterized by the presence of endothelial damage or denudation without actual fibrous cap disruption [18,42,43]. These plaques tend to have a composition rich in smooth muscle cells and extracellular matrix, with a lower prevalence of lipid or necrotic cores [26,42,44]. Erosions are particularly more common in younger individuals and women and are often associated with local inflammation, neutrophil infiltration, and endothelial apoptosis [27,42]. The third pathological mechanism, eruptive CNs, although less frequent, are characterized by protruding, fragmented calcified structures that penetrate the fibrous cap and result in thrombosis [3,26,27]. These lesions are more prevalent in older patients and those with chronic kidney disease [27]. The recognition of these diverse mechanisms has led to the understanding that the concept of vulnerable plaque must include all these substrates, given their shared potential to precipitate ACS.

A pivotal concept discussed in the review is the importance of plaque burden. Evidence suggests that, beyond specific morphological characteristics, the overall burden of atherosclerosis significantly influences the risk of adverse cardiovascular events. Studies such as PROSPECT [33] and VIVA [34] have demonstrated that a higher plaque burden is a strong predictor of future events. For instance, lesions with an IVUS-derived plaque burden of 70% or greater were associated with a significantly higher incidence of MACE. However, plaque burden alone does not fully predict risk. In PROSPECT II [20], plaques with ≥70% burden were only considered high-risk if they were also lipid-rich as defined by NIRS. Moreover, the authors emphasize that plaque burden is not only a determinant of mechanical obstruction but also reflects the systemic extent of the disease, contributing to a higher probability of lesion destabilization. The almost linear correlation between total CAD burden and adverse events suggests that larger disease burdens increase the probability of plaque destabilization and subsequent clinical events [8]. This reinforces the need to consider both specific plaque features and the overall extent of atherosclerosis for accurate risk stratification. However, the presence of high-risk plaque features does not invariably translate into clinical events, as demonstrated by the concept of silent plaque disruption and healing [9]. Increasing evidence indicates that many plaques undergo subclinical rupture or erosion followed by healing, without manifesting as symptomatic or clinically evident episodes. Histopathological and imaging studies have identified healed plaques, particularly in patients with chronic coronary syndromes [112]. These healed plaques, often characterized by layered structures within the intima [3,113,114], suggest a dynamic and cyclical process of plaque destabilization and repair. This silent evolution of plaques complicates risk assessment and underscores the importance of longitudinal monitoring. Moreover, the progression from a silent plaque rupture to an acute event depends on several factors, including the degree of luminal compromise, the extent of thrombosis, and the patient’s systemic inflammatory status [115,116].

The accurate detection of high-risk plaques in vivo remains a critical challenge and relies on advanced imaging modalities. Invasive imaging techniques such as OCT and IVUS have provided invaluable insights into plaque morphology. OCT, with its high spatial resolution, is particularly effective in detecting thin fibrous caps, macrophage infiltration, microvessels, allowing for detailed assessment of TCFA characteristics [3,4]. IVUS, including its virtual histology variant (IVUS-VH), offers robust evaluation of plaque burden and composition, enabling the identification of lipid cores and fibrous tissue [117]. NIRS adds another layer of precision by enabling the identification of lipid-rich plaques through the detection of their chemical signatures [20,21]. This modality is particularly valuable for assessing LCBI, which quantifies the extent of lipid accumulation within plaques. In the PROSPECT II trial [20], it was demonstrated that plaques with ≥70% burden were not considered high-risk unless they also displayed lipid-rich features identified by NIRS. This emphasizes the crucial role of lipid content, in addition to plaque size, in defining plaque vulnerability.

Non-invasive imaging, particularly Coronary Computed Tomography Angiography (CCTA), plays an essential role in the detection of high-risk plaques and has evolved to provide insights beyond simple anatomical assessment. CCTA has become essential in the non-invasive assessment of CAD, providing detailed anatomical evaluation of the coronary lumen and vessel wall. Technological advancements have enhanced its accuracy, allowing not only the detection of stenosis but also the assessment of plaque composition and vascular inflammation [118,119]. A key strength of CCTA is its ability to identify vulnerable plaque features—including low-attenuation plaque, positive remodeling, spotty calcifications, and the napkin-ring sign—making it valuable for both diagnostic and prognostic purposes [120]. Unlike traditional imaging focused on stenosis severity, CCTA offers insights into plaque vulnerability, a major determinant of cardiovascular risk. Studies like ICONIC [121], SCOT-HEART [122], and PROMISE [123] confirm that plaque characteristics, beyond luminal narrowing, are strong predictors of adverse cardiovascular events. Particularly, the SCOT-HEART trial provided robust evidence for the prognostic value of CCTA, showing a significant reduction in cardiovascular death and non-fatal MI when CCTA was used in addition to standard testing [124]. Despite challenges such as spatial resolution limitations and coronary calcifications, CCTA complements intravascular imaging modalities (IVUS, OCT, NIRS) in defining plaque phenotype and vulnerability. Moreover, unlike invasive techniques limited to specific vessel segments, CCTA provides a more comprehensive evaluation of plaque burden and morphology. A groundbreaking advancement in CCTA imaging is its ability to assess coronary inflammation through perivascular Fat Attenuation Index (FAI) [119], a CCTA-derived biomarker, able to quantify spatial gradients in perivascular adipose tissue (PVAT) attenuation, serving as an indicator of underlying vascular inflammation [119,125]. PVAT, once considered a passive structural component, is now recognized as an active regulator of vascular homeostasis, influencing inflammation, vascular tone, and metabolic processes through the secretion of bioactive molecules. Positioned around blood vessels, PVAT engages in bidirectional cross-talk with the vascular wall, making it both a therapeutic target and a diagnostic and prognostic biomarker in cardiovascular disease [119,125]. The CRISP-CT study [126], which analyzed data from 3912 patients, demonstrated that elevated perivascular FAI is a strong predictor of cardiac mortality and MACE, independent of traditional risk factors and plaque burden. Notably, the study also revealed that FAI values decrease after statin therapy initiation, underscoring its potential as a monitoring tool for therapeutic response.

Importantly, CCTA has emerged not only as a key tool for detecting plaque morphology but also for quantifying the amount of myocardium at risk through advanced computational methods. This capability provides critical information on the potential clinical implications of plaque destabilization. In their review, the authors introduce a particularly insightful concept: the assessment of whether a plaque is truly “high-risk” should extend beyond traditional metrics like vulnerability features and plaque burden. It should also incorporate the anatomical context—specifically, the amount of myocardium at risk supplied by the artery harboring the lesion [8].

This approach acknowledges that the clinical consequences of PR or PE are not solely determined by the intrinsic characteristics of the lesion but are also profoundly influenced by its anatomical location. Therefore, a plaque’s location—and the extent of myocardium it puts at risk—becomes a fundamental factor in defining its overall clinical risk. The authors also emphasize the importance of non-invasive imaging modalities such as positron emission tomography (PET) and CMR. PET, particularly with tracers like 18F-sodium fluoride, allows for the detection of active calcification and inflammation, identifying biologically active plaques at higher risk of progression. CMR, although limited in coronary imaging, provides detailed characterization of myocardial tissue, enabling the assessment of fibrosis, edema, and scar formation. These techniques offer complementary information on plaque activity and myocardial health, enhancing overall risk stratification [8]. Each imaging modality offers unique and complementary insights into the comprehensive assessment of high-risk plaques. OCT excels at characterizing plaque microstructure, enabling detailed visualization of fibrous cap thickness, lipid-rich core, macrophage infiltration, and all major substrates contributing to ACS. IVUS is particularly valuable for evaluating overall plaque burden and vessel remodeling, while NIRS provides precise identification of lipid-rich plaques by detecting their chemical composition. CCTA plays a crucial role in the non-invasive assessment of plaque morphology, burden, and inflammation, including the quantification of perivascular fat and myocardium at risk. PET adds a functional perspective by detecting active calcification and inflammation, identifying plaques with a higher likelihood of progression. Lastly, CMR contributes essential information on myocardial tissue characterization, assessing fibrosis, edema, and scarring. The integration of these modalities, combining morphological, functional, and anatomical data, is fundamental for accurate risk stratification and personalized management of patients with CAD.

In conclusion, the revised concept of vulnerable plaque advocates for a holistic approach that integrates multiple dimensions of plaque assessment, including morphology, biological activity, systemic risk factors, and lesion-specific characteristics. Early detection, aggressive medical therapy, and, when appropriate, interventional strategies are fundamental to mitigating the risk of acute cardiovascular events. This comprehensive perspective reflects the complexity of atherosclerosis and emphasizes the need for continuous refinement of diagnostic and therapeutic strategies in cardiovascular medicine. The insightful state-of-the-art review by Vergallo et al. represents a significant advancement in modern cardiovascular medicine. By redefining the concept of high-risk plaque and introducing innovative considerations—such as the role of myocardium at risk and the integration of advanced imaging modalities—this work provides a robust framework for future research and clinical practice. It underscores the necessity of a multidimensional approach to risk assessment and management, encouraging the adoption of refined, patient-centered strategies to better predict, prevent, and treat acute coronary events.

Despite the significant advancements in cardiovascular imaging technologies, it is important to remember that laboratory biomarkers remain the cornerstone of first-line diagnostic evaluation in ischemic heart disease. These parameters provide immediate, accessible, and highly informative data that complement imaging findings and help guide early clinical decision-making. Among these, cardiac troponins—particularly high-sensitivity Troponin T—are fundamental for the diagnosis of acute myocardial infarction and play a pivotal role in distinguishing between type 1 and type 2 myocardial infarction in patients with MINOCA. Similarly, NT-proBNP serves as a reliable marker of myocardial wall stress and dysfunction, contributing to both diagnostic accuracy and prognostic stratification in acute and chronic coronary syndromes [127]. When interpreted alongside advanced imaging techniques such as OCT, CCTA, or CMR, these biomarkers enhance diagnostic precision by providing functional and biological context to anatomical findings. Therefore, rather than viewing laboratory and imaging modalities as isolated tools, they should be considered complementary components of an integrated, patient-centered approach to ischemic heart disease evaluation and management.

### 6.2. Plaque Healing

**Figure 5:**
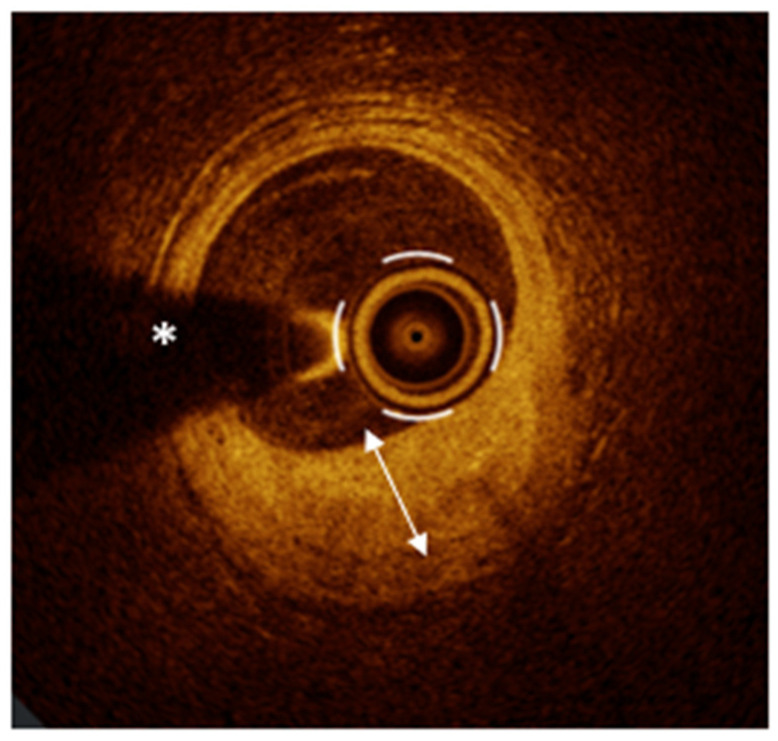
Healed plaque. Layered structure with an “onion-like” appearance, featuring one or more layers with intense, heterogeneous signals layer of different optical densities (double-headed arrow) and a distinct demarcation from the underlying tissue. *The asterisk is indicating the guide wire artifact. All images come from the authors’ personal archive, unless otherwise indicated.*

In recent years, the concept of “plaque healing” has attracted considerable attention with the “double-hit theory” emerging as a framework for understanding ACS development. This framework suggests that plaque destabilization alone is not sufficient to trigger an acute event; rather, it is the combination of plaque disruption and an inadequate healing response that leads to clinical manifestation. Indeed, effective healing mechanisms can stabilize a disrupted plaque, allowing it to “self-repair” and preventing progression to an acute coronary syndrome [9]. Through OCT, a “healed” plaque typically exhibits a layered, “*onion-like*” structure with one or more layers of intense, heterogeneous signal, sharply demarcated from the underlying tissue (Figure 5) [3,113,114].

Multiple studies have investigated the role of healed plaques to better define their clinical significance. These studies have shown that healed plaques are more frequently observed in patients with CCS than in those with ACS [112], and can also be a common finding in ACS, involving both culprit and non-culprit lesions. Moreover, patients with ACS displaying a “layered” pattern at culprit sites often exhibit similar features at non-culprit sites as well [115]. This evidence suggests that while plaque healing represents a mechanism of stabilization, it also serves as a marker of previous plaque destabilizations over time, providing an indirect measure of overall disease vulnerability. In line with this concept, recent studies have highlighted the significance of layered plaques as indicators of prior plaque destabilization. A study by Russo et al. [116] analyzing ACS patients with pre-intervention OCT imaging found that 28.4% had layered culprit plaques, linked to greater macrophage infiltration at non-culprit sites, suggesting widespread coronary vulnerability rather than a localized process. Additionally, irrespective of culprit plaque phenotype, layered non-culprit plaques exhibited greater lipid content, higher prevalence of TCFA, and increased macrophage infiltration, all features of plaque vulnerability. These findings align with those observed by Fracassi et al. [10], who investigated healed plaques in ACS patients using OCT, finding that 28.7% of 376 patients had healed plaques, more often in individuals with diabetes, hyperlipidemia, and prior MI. These patients showed higher systemic inflammation (elevated hs-CRP) and plaques with more frequent PR, TCFA, macrophage infiltration, and greater stenosis. Although MACE rates were similar, all-cause rehospitalization was higher in patients with healed plaques, suggesting that while healing may temporarily stabilize lesions, the presence of a layered phenotype reflects persistent vulnerability driven by both local and systemic inflammation, predisposing to future events. Expanding on this concept, a recent study [128] on serial OCT imaging explored the clinical significance of newly formed layered patterns in non-culprit plaques over time. The study demonstrated that TCFA, macrophage infiltration, and thrombus were independent predictors of new layered plaque formation. Over a one-year follow-up, plaques with new layered patterns showed a progressive decrease in luminal area, a reduction in lipid content, and an increase in fibrous cap thickness. These changes indicate that while plaque healing initially stabilizes the plaque, it simultaneously contributes to progressive luminal stenosis, potentially increasing the risk of future adverse cardiovascular events. These studies underscore the dual nature of plaque healing. While it may contribute to plaque stabilization and lower the risk of MACE, it also acts as an indicator of coronary vulnerability, with the layered morphology reflecting previous episodes of plaque destabilization. Notably, plaque healing is linked to a progressive luminal narrowing, a recognized predictor of cardiovascular adverse events [32].

### 6.3. Myocardial Bridge

MB is a congenital coronary anomaly characterized by an intramyocardial segment of an epicardial coronary artery, most commonly affecting the left anterior descending artery (LAD) [129]. The prevalence of MB varies significantly depending on the imaging modality: 2–6% on coronary angiography, 19–22% on CCTA and up to 33–42% in autopsy studies [130]. This intramyocardial course results in dynamic compression of the artery during the cardiac cycle, particularly during systole, and can lead to AMIS and NAMIS [2,24,101,130]. The hallmark of MB is the systolic compression of the tunneled artery, known as the “*milking effect*,” which is typically visualized on coronary angiography [131]. While systolic compression alone was initially considered hemodynamically insignificant—since coronary perfusion predominantly occurs during diastole—it is now recognized that MB can impair early diastolic blood flow. This occurs because the tunneled artery often exhibits delayed diastolic relaxation, leading to sustained compression that impairs coronary perfusion during the critical phase of early diastolic filling [130]. The severity of ischemia induced by MB depends on various anatomical and physiological factors, including the depth, length, and thickness of the overlying myocardium. Deeper bridges (≥2 mm) are associated with more pronounced systolic compression and greater ischemic potential, while longer MB segments can lead to more extensive ischemic territories. Greater thickness of the overlying myocardium increases the external compressive force exerted on the coronary artery [130]. Although the main obstruction occurs during systole, studies have shown that MB can impair diastolic coronary flow as well. The delayed relaxation of the myocardium can prolong the period of vessel compression into early diastole, reducing coronary perfusion, particularly in the context of increased heart rates where diastolic filling time is shortened. This impairment is often detected by invasive physiological tests like diastolic fractional flow reserve (dFFR), particularly when performed under pharmacological stress [132]. Another mechanism contributing to ischemia in MB is the “branch steal phenomenon”, where septal or diagonal branches originating within or proximal to the bridged segment experience reduced perfusion. During systolic compression, the preferential diversion of blood flow away from these branches can lead to localized ischemia in the myocardial territories they supply. This phenomenon is especially significant in deeper and longer MBs, where side branches are more likely to be affected. The ischemia resulting from branch steal is often underestimated, yet it can significantly contribute to anginal symptoms and impaired myocardial perfusion [133]. The unique biomechanical forces exerted on the arterial wall by MB play a significant role in the pathogenesis of atherosclerosis. The segment of the artery proximal to the MB is exposed to low wall shear stress (WSS) and high oscillatory flow patterns, which are known to promote endothelial dysfunction and atherosclerotic plaque development [134,135,136]. In contrast, the tunneled segment of the artery is subjected to high WSS, which appears to exert a protective effect against the development of atherosclerosis [134]. Additionally, the absence of adventitial VV within the bridged segment, as identified by OCT imaging [137], may contribute to its resistance to plaque formation. The lack of VV limits the nutrient supply to the vessel wall, which may reduce the propensity for neovascularization and subsequent atherosclerotic infiltration. MB is often associated with endothelial dysfunction, particularly in the proximal segment of the artery [101,102]. This dysfunction results in impaired vasodilation and an exaggerated vasoconstrictive response to stimuli such as Ach. This vasospastic component can contribute to transient episodes of myocardial ischemia, even in the absence of significant dynamic compression. Furthermore, endothelial dysfunction may extend into the bridged segment, where abnormal vasomotor responses can lead to vasospasm. Vasospasm superimposed on systolic compression can exacerbate ischemia and worsen clinical outcomes [130]. Finally, the interplay between systolic compression, impaired diastolic relaxation, branch steal phenomenon, proximal atherosclerosis, and vasomotor dysfunction underlines the complexity of MB and necessitates a comprehensive diagnostic and therapeutic approach. In summary, MB is a dynamic condition influenced by anatomical factors, coronary hemodynamics, and vascular biology.

The diagnosis of MB requires an integrated approach utilizing both invasive and non-invasive imaging modalities to achieve accurate detection and characterization. Coronary angiography remains the conventional approach for the detection of MB. It typically reveals the characteristic “*milking effect*”, which manifests as systolic compression of the coronary artery with subsequent relaxation during diastole [131]. However, the sensitivity of coronary angiography is limited as many MBs, particularly those that are shallow or not associated with significant dynamic compression, may go undetected [130]. This limitation has led to the growing importance of intravascular imaging techniques such as OCT and IVUS. IVUS has proven to be a valuable tool in the detailed morphological assessment of MB. It identifies MB through the visualization of the “*half-moon*” sign, which is an echolucent area corresponding to the muscle overlying the artery. This imaging feature allows for a more accurate evaluation of the depth, length, and thickness of the MB [130,131]. Despite these advantages over coronary angiography, IVUS lacks the ability to assess the functional significance of MB, such as its dynamic effects on coronary blood flow under varying physiological conditions. Functional evaluation of MB is critical to determine its hemodynamic impact and guide management decisions. FFR and dFFR are essential tools for this purpose. FFR, performed during hyperemia, helps quantify the degree of flow limitation caused by MB [130], while dFFR provides a more sensitive assessment, particularly under stress conditions induced by agents like dobutamine. dFFR is considered superior in capturing the diastolic impact of MB, especially since coronary perfusion predominantly occurs during diastole [132]. Non-invasive imaging techniques also play a significant role in MB assessment. CCTA offers high anatomical resolution and is effective in identifying the depth, length, and angulation of MB. It is particularly useful in initial evaluations and longitudinal follow-ups. However, CCTA has limited ability to assess the dynamic aspects of MB, such as its functional impact on coronary flow during stress conditions. Therefore, while it is valuable for anatomical assessment, it must often be complemented with functional testing to fully understand the clinical significance of MB [130].

**Figure 6:**
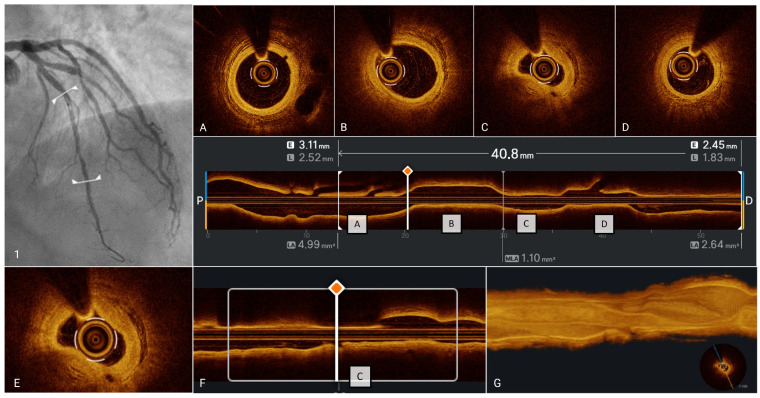
Myocardial bridge. Clinical case of a 74-year-old man with rest angina who underwent coronary angiography. Panel (**1**): coronary angiography revealed a myocardial bridge of the mid-LAD with the classic “milking effect,” which manifests as systolic compression of the coronary artery with subsequent relaxation during diastole. To better evaluate the bridge and assess for concomitant coronary stenosis, an OCT pullback was performed (Panels (**A**–**D**)), showing a long myocardial bridge in the mid-to-distal LAD with the characteristic appearance of “moon-shaped” areas surrounding the adventitia with well-demarcated borders, and a heterogeneous, middle-backscattering signal (dashed curved line). All segments showed a normal three-layered vessel structure with no features of atherosclerosis. Panels (**E**,**F**): magnification of the segment most compressed by the bridge, demonstrating significant luminal narrowing with a minimal lumen area of 1.10 mm^2^, shown in both cross-sectional and longitudinal views, with “bumps” and intimal folding caused by external systolic compression. Panel (**G**): 3D reconstruction of the same vessel segment showing bumps and depressions of the intima, generated by intimal folding during systole due to external compression. *All images come from the authors’ personal archive, unless otherwise indicated.*

OCT offers a superior, high-resolution imaging modality that enables the detection of structural features often missed by other techniques. It identifies MB as a moon-shape area with intermediate light intensity surrounding the adventitia with demarked borders and heterogeneous, middle-backscattering signal (Figure 6) [138]. This OCT-defined appearance has been validated against IVUS findings, where MB typically presents as a “half-moon” sign, characterized by an echolucent, crescent-shaped area surrounding the artery during systole [129,131,138]. OCT is highly effective in measuring the depth, thickness, and angular extent of the MB, offering critical data to assess the anatomical complexity of the MB [130]. Moreover, OCT provides a clear and detailed visualization of the vessel within the intramyocardial segment (generally protected from atherosclerosis due to conditions of high shear stress) and immediately proximal and distal to the bridged segment, prone to developing atherosclerosis due to conditions of low shear stress and altered flow dynamics [134,135,136]. Furthermore, the absence of adventitial VV in the bridged segment can be identified through OCT imaging, contributing to a better understanding of the vessel’s unique biological behavior and its relative resistance to atherosclerosis [137].

#### OCT Ad Guidance for PCI in Myocardial Bridge

The treatment strategy for MB primarily depends on the severity of symptoms, anatomical characteristics, and the patient’s response to initial medical therapy. First-line management is pharmacological, aimed at alleviating symptoms and reducing myocardial oxygen demand. Medical therapy for MB primarily involves beta-blockers to reduce heart rate and alleviate symptoms, with calcium-channel blockers or ivabradine as alternatives. Nitrates are generally avoided as they can worsen systolic compression. Surgical options, such as myotomy or coronary artery bypass grafting (CABG), are reserved for severe, refractory cases but carry higher risks and require careful patient selection [130]. For patients with persistent symptoms despite optimized medical therapy, PCI can be considered [130]. However, PCI in MB presents unique challenges. The mechanical forces exerted by the overlying myocardial fibers increase the risk of complications, including stent fracture, in-stent restenosis (ISR), and coronary perforation. The use of second-generation DES with high radial strength has shown some benefit in reducing ISR rates, though it does not eliminate the risk entirely.

Careful intravascular imaging with IVUS or OCT is essential for optimal stent selection and precise deployment. Accurate sizing and placement are crucial to avoid complications and ensure long-term stent patency. Despite these strategies, ISR remains a considerable concern, particularly in severe MBs characterized by longer and deeper segments [130]. Finally, in the context of MB, OCT is not only instrumental in diagnostic assessment but also serves as guidance for PCI. It allows for the optimization of stent selection, sizing, and placement, minimizing the risk of complications such as ISR and stent malapposition [6]. The precision of OCT in evaluating post-stent expansion and apposition ensures that the stent is properly deployed, reducing the likelihood of future procedural failure. OCT also aids in identifying potential procedural risks, such as coronary perforation, by providing detailed insights into vessel wall thickness and the relationship of the stent to the surrounding myocardial tissue [130]. Additionally, OCT can highlight the interaction between the stent and the dynamic nature of the myocardial bridge, allowing for real-time adjustments to the interventional strategy if necessary. Its ability to detect even minor discrepancies post-stenting is crucial for improving long-term procedural outcomes [130]. In this scenario, a retrospective analysis by T. Xu et al. [139] emphasized the importance of OCT in evaluating MB characteristics and monitoring outcomes following DES implantation. The study confirmed that the visible muscle layer surrounding the vessel adventitia, as detected by OCT, corresponded to the “*half-moon*” layer seen on IVUS. The analysis also demonstrated that the severity of MB, characterized by the thickness and extent of the muscle layer, correlated with an increased risk of ISR. Notably, ISR rates were significantly higher in patients with severe MB compared to those with mild or no MB. These findings highlight the need for careful consideration when planning PCI in MB segments, as the structural characteristics of MB may predispose to higher ISR rates. In conclusion, OCT has emerged as a pivotal tool in the diagnosis and management of MB. It provides high-resolution visualization of the intramyocardial segment, typically protected from atherosclerosis due to high shear stress, and of the immediately proximal segments, which are often affected by atherosclerosis due to low shear stress. OCT is highly effective in measuring the depth, thickness, and angular extent of the MB, closely linked to the degree of ischemia resulting from MB. This detailed evaluation is essential for determining the severity of dynamic compression and for planning precise interventional strategies. OCT is also essential during PCI, guiding optimal stent selection, sizing, and placement, and minimizing risks such as ISR and coronary perforation. By offering precise anatomical and procedural insights, OCT enhances both diagnostic accuracy and interventional outcomes, solidifying its role in the comprehensive management of MB.

## 7. Beyond the Potential of OCT: The Role of Functional Coronary Assessment for a Definitive Diagnosis in AMIS and NAMIS with No-Obstructive Coronary Arteries

As previously discussed, CMD has emerged as a pivotal mechanism contributing to both AMIS and NAMIS [2]. Growing evidence indicates that CMD plays a central role in MINOCA and INOCA [140]. The mechanisms through which CMD contributes to AMIS and NAMIS differ significantly. In AMIS, transient or persistent microvascular dysfunction, often resulting from inflammation, endothelial dysfunction, or microembolization, impairs vasodilation or induces microvascular spasm, ultimately leading to myocardial ischemia and infarction without obstructive coronary disease. CMD in this context is frequently associated with conditions such as MINOCA and Takotsubo syndrome, where impaired microvascular perfusion exacerbates myocardial injury [141,142]. Conversely, in NAMIS, CMD manifests as impaired coronary blood flow regulation due to increased microvascular resistance, reduced vasodilatory capacity, or microvascular spasm [143]. Patients with INOCA commonly exhibit CMD, which contributes to exertional angina, reduced exercise tolerance, and diminished quality of life.

Given the heterogeneity in CMD pathophysiology, a comprehensive diagnostic approach extending beyond structural imaging is essential for accurate ischemia assessment. While OCT is valuable for detecting intraluminal abnormalities and vessel wall characteristics, it is inadequate for evaluating functional and dynamic coronary disorders, underscoring the need for an integrative diagnostic approach. The *#FullPhysiology* (#FP) algorithm has emerged as a promising approach for this purpose [87]. This systematic, invasive, stepwise protocol evaluates each component of coronary circulation, including epicardial, microvascular, and vasomotor function. It is performed in the catheterization laboratory using a dedicated guidewire (Pressure Wire X, Abbott, North Chicago, IL, USA) combining pressure and flow measurements. The process begins with the assessment of epicardial disease using FFR. Then, microvascular compartment is assessed using CFR and IMR. CFR quantifies the ability of coronary circulation to augment blood flow in response to metabolic demand, with values below 2.0 indicating impaired vasodilatory function. IMR measures microcirculatory resistance during hyperemia, with values exceeding 25 defining abnormal microvascular function. Importantly, the epicardial and microvascular domains should be considered as a continuum rather than distinct entities. Recent research underscores the complex interplay between FFR and IMR, demonstrating that microvascular dysfunction can influence FFR measurements, particularly when IMR exceeds 40 [144]. This finding reinforces the necessity of evaluating both epicardial and microvascular compartments for accurate ischemic burden assessment and optimized patient management. Ach provocation testing represents a crucial final component of the #FP approach, facilitating the identification of epicardial and microvascular spasm based on the Coronary Vasomotor Disorders International Study Group (COVADIS) criteria [145].

The #FP approach is instrumental in guiding individualized treatment strategies and prognostic assessment in AMIS and NAMIS. In AMIS, identifying microvascular spasm as a marker of microvascular dysfunction supports the use of vasodilatory agents such as calcium channel blockers and nitrates, which enhance endothelial function and mitigate spasm-related ischemia. Additionally, IMR assessment during the acute myocardial infarction phase aids in stratifying patients at risk of persistent microvascular dysfunction and adverse outcomes warranting targeted pharmacological interventions [146].

In NAMIS, the #FP approach enables precise classification of INOCA endotypes, thereby facilitating targeted therapy and improving clinical outcomes [143]. CFR and IMR evaluations categorize CMD into two primary subtypes [147]. Structural CMD, characterized by reduced CFR and elevated IMR, could benefit from therapies aimed at reducing microvascular resistance, such as renin–angiotensin–aldosterone system inhibitors and statins [147]. Functional CMD, defined by reduced CFR but normal IMR, responds favorably to endothelial-modulating agents such as beta-blockers and lifestyle modifications [147]. ACh testing further aids in identifying microvascular spasm, guiding the use of calcium channel blockers or nitrates [143]. Recent studies and meta-analyses underscore the strong correlation between reduced CFR and adverse cardiovascular outcomes [148,149]. Moreover, the CORMICA trial demonstrated that tailored medical therapy based on INOCA endotypes led to improved symptom control at follow-up [150]. These findings have influenced the latest 2024 guidelines on chronic coronary syndromes, which now grant a Class I, Level B recommendation for functional testing in symptomatic patients with non-obstructive CAD [22]. Additionally, the recent CHAMP-CMD study confirmed that only patients with reduced CFR derive benefit from tailored therapy in the INOCA setting [151]. Furthermore, a real-world study by Leone et al. demonstrated that implementing the #FP approach in patients with non-functionally relevant intermediate epicardial stenoses resulted in reduced cardiac hospitalizations, lower healthcare costs, and improved symptom control after one year [152].

These findings support the integration of complementary imaging and physiological modalities for a definitive diagnosis and personalized management of patients with AMIS and NAMIS. By providing a comprehensive evaluation of coronary function, the #FP approach enables precise risk stratification, guides tailored therapeutic strategies, and ultimately improves clinical outcomes in this challenging patient population.

Nevertheless, despite the strong value of functional testing, there are clinical scenarios in which OCT should be prioritized. In acute settings such as MINOCA (type 1), where patients present with AMI and non-obstructive coronary arteries, the underlying pathophysiology often involves non-flow-limiting PR or PE, intracoronary thrombus, or SCAD—conditions that may not cause measurable flow abnormalities. In these cases, FFR or iFR may be uninformative or misleading, and OCT’s superior spatial resolution becomes essential for detecting subtle endoluminal alterations, such as fibrous cap disruption, thrombus, or intramural hematoma. Similarly, OCT should be favored in situations where angiographic findings are ambiguous—such as hazy or ulcerated lesions, overlapping arterial segments, or aneurysmal dilatations—where functional measurements may not clarify the diagnosis. In such contexts, OCT offers precise structural insights that can identify the culprit lesion, rule out plaque disruption, or confirm a diagnosis such as SCAD, particularly when visualizing an intimal flap or entry point of dissection.

In conclusion, while functional testing remains indispensable for evaluating ischemia in patients with ambiguous epicardial disease and suspected CMD, OCT plays a unique and irreplaceable role in uncovering structural abnormalities that are not detectable by pressure-based indices. A nuanced, scenario-driven approach that integrates both functional and anatomical tools is essential for the accurate diagnosis and management of ischemia with non-obstructive coronary arteries.

## 8. Conclusions

In the evolving landscape of ischemic heart disease, the distinction between AMIS and NAMIS represents a pivotal conceptual shift, moving from an “anatomy-based” to a “pathophysiology-centered” framework. This new classification embraces the full complexity of ischemic mechanisms—both obstructive and non-obstructive, acute and non-acute—enhancing diagnostic precision and paving the way for personalized therapeutic strategies. Within this paradigm, OCT emerges as a transformative tool, capable of shedding light on the subtle nuances of coronary pathology that conventional angiography may leave in shadow. In AMIS, OCT enables the identification of distinct mechanisms of plaque destabilization—including PR, PE, and eruptive CN—as well as characterizing the composition and burden of intracoronary thrombi. In cases of MINOCA, OCT provides invaluable insights that distinguish between atherosclerotic and non-atherosclerotic causes such as SCAD, coronary embolism, and epicardial vasospasm, thereby refining diagnosis and guiding treatment. In NAMIS, OCT plays a central role in evaluating both the severity and morphological characteristics of epicardial coronary stenoses, allowing for the identification of key features of plaque vulnerability and evidence of plaque healing. By doing so, it provides valuable insights into the natural history of atherosclerosis, offering a deeper understanding of its dynamic and evolving nature. Moreover, OCT represents a valuable adjunct in the assessment of conditions such as MB and epicardial vasospasm—mechanisms that may elude detection through conventional imaging. Although OCT is not the first-line modality for diagnosing these entities, it offers unique structural and anatomical insights that enhance our understanding of their pathophysiology and clinical relevance.

Looking ahead, several key areas warrant further investigation. There is a clear need for large-scale, prospective studies to evaluate the prognostic implications of OCT-defined plaque features, particularly in patients with non-obstructive ischemia. Future research should also assess how OCT can be integrated into multimodal diagnostic pathways, combining morphological data with functional assessments such as FFR/iFR or non-invasive imaging. Moreover, the development of artificial intelligence tools for real-time OCT interpretation could significantly improve workflow and expand access to this modality beyond tertiary centers. Finally, randomized clinical trials are needed to test whether OCT-guided treatment strategies, particularly in non-culprit or angiographically silent lesions, translate into improved clinical outcomes. In this context, OCT holds not only diagnostic and therapeutic value but also the potential to shape the future of precision cardiology.

Just as the prisoner in Plato’s cave begins to understand the world only after emerging into the light, so too does OCT allow clinicians to transcend the shadows of traditional imaging, uncovering a richer, more nuanced reality of coronary pathophysiology. By “*removing the blindfold*”, OCT does not merely reveal what was hidden—it transforms how we see, think, and treat ischemic heart disease. As we continue to integrate OCT into the diagnostic algorithm for ischemic syndromes, its role evolves from supportive to essential: a tool not merely of precision, but of revelation. It guides us beyond simplistic models, illuminating the rich, multifaceted nature of CAD—and in doing so, it leads us not only toward greater clinical clarity, but toward a deeper appreciation of the beauty within complexity.

## Figures and Tables

**Table 1:** OCT Diagnostic roles across ischemic syndromes.

Acute Myocardial Ischemic Syndromes		
Syndrome	Mechanism	OCT Diagnostic Role
Acute Coronary Syndrome	Flow-limiting plaque rupture, plaque erosion, eruptive calcified nodule.	Characterizes culprit lesions; identifies mechanism of acute coronary syndromes; characterizes thrombus burden and type; assess underlying plaque phenotype and vulnerability.
Myocardial Infarction with Non-Obstructive Coronary Artery Disease type 1	Non-flow limiting plaque rupture, plaque erosion, eruptive calcified nodule.	Characterizes culprit lesions; identifies mechanism of plaque destabilization; characterizes thrombus burden and type; assess underlying plaque phenotype and vulnerability.
Myocardial Infarction with Non-Obstructive Coronary Artery Disease type 2	Spontaneous coronary artery dissection, coronary embolism, epicardial spasm	Identifies intimal flap and/or intramural hematoma; visualizes thrombus without underlying plaque; confirms vasospasm-related changes in vessel architecture.
Non-Acute Myocardial Ischemic Syndromes		
Epicardial stenoses	Atherosclerotic plaque with different plaque phenotype	Assesses plaque phenotype and severity; identifies vulnerable plaque features such as thin fibrous cap, large lipid pool, macrophages, microchannels, cholesterol crystal and “plaque healing”.
Ischemia with Non-Obstructive Coronary Arteries	Epicardial spasm, myocardial bridge	Confirms vasospasm-related changes in vessel architecture; visualizes ‘half-moon’ appearance caused by muscle overlying the artery.

## Data Availability

All data generated or analyzed during this study are included in this published article. Further inquiries should be directed to the corresponding author.

## Abbreviations

The following abbreviations are used in this manuscript:

AMIS	Acute Myocardial Ischemic Syndromes
NAMIS	Non-Acute Myocardial Ischemic Syndromes
OCT	Optical Coherence Tomography
CAD	Coronary artery disease
ACS	acute coronary syndromes
PCI	Percutaneous Coronary Intervention
PR	Plaque rupture
PE	Plaque erosion
CN	Calcified nodule
MINOCA	Myocardial Infarction with Non-Obstructive Coronary Arteries
SCAD	Spontaneous coronary artery dissection
INOCA	Ischemia with Non-Obstructive Coronary Arteries
MB	Myocardial bridges
IVUS	Intravascular ultrasound
TD-OCT	Time-domain OCT
OFD	Optical frequency domain
IVI	Intravascular imaging
AI	Artificial intelligence
NIRS	Near-infrared spectroscopy
CCS	Chronic coronary syndromes
LM	Left main
CMVD	Coronary microvascular dysfunction
CFT	Coronary functional tests
TCFA	Thin-cap fibroatheroma
MACE	Major adverse cardiac event
MI	Myocardial infarction
BVS	Bioresorbable vascular scaffold
MLA	Minimal lumen area
QCA	Quantitative coronary angiography
DES	Drug-eluting stent
ST	Stent thrombosis
ARC	Academic Research Consortium
DAPT	Dual antiplatelet therapy
AMI	Acute myocardial infarction
LM	Left main
SED	Stent edge dissection
MSA	Minimum stent area
EAPCI	European Association of Percutaneous Cardiovascular Intervention
SM	Stent malapposition
ASM	Acute stent malapposition
LSM	Late stent malapposition
TMV	Total malapposition volume
CMR	Cardiac magnetic resonance
LCBI	Lipid core burden index
TL	True lumen
FL	False lumen
EEL	External elastic lamina
VV	Vasa vasorum
ECG	Electrocardiogram
Ach	Acetylcholine
VSA	Vasospastic angina
FFR	Fractional Flow Reserve
IFR	Instantaneous Wave-Free Ratio
MLD	Minimal lumen diameter
AICLs	Angiographically intermediate coronary lesions
AS	Area stenosis
OFR	Optical Flow Ratio
CCs	Cholesterol crystals
VSMC	Vascular smooth muscle cell
MFR	Myocardial flow reserve
MPR	Myocardial perfusion reserve
NHPRs	Non-hyperemic pressure ratios
RFR	Resting Full-cycle Ratio
ANOCA	Angina with No Obstructive Coronary Artery disease
IMR	Index of Microcirculatory Resistance
IVUS-VH	IVUS virtual histology
CCTA	Coronary computed tomography angiography
FAI	Fatty attenuation index
PVAT	Perivascular adipose tissue
PET	Positron emission tomography
LAD	Left anterior descending artery
dFFR	Diastolic FFR
WSS	Wall shear stress
ISR	In-stent restenosis
CABG	Coronary artery bypass grafting
CFR	Coronary flow reserve
IMR	Index of Microcirculatory Resistance
